# Introns provide a platform for intergenic regulatory feedback of *RPL22* paralogs in yeast

**DOI:** 10.1371/journal.pone.0190685

**Published:** 2018-01-05

**Authors:** Kateřina Abrhámová, Filip Nemčko, Jiří Libus, Martin Převorovský, Martina Hálová, František Půta, Petr Folk

**Affiliations:** Department of Cell Biology, Faculty of Science, Charles University, Prague, Czech Republic; University of Toronto, CANADA

## Abstract

Ribosomal protein genes (RPGs) in *Saccharomyces cerevisiae* are a remarkable regulatory group that may serve as a model for understanding genetic redundancy in evolutionary adaptations. Most RPGs exist as pairs of highly conserved functional paralogs with divergent untranslated regions and introns. We examined the roles of introns in strains with various combinations of intron and gene deletions in *RPL22*, *RPL2*, *RPL16*, *RPL37*, *RPL17*, *RPS0*, and *RPS18* paralog pairs. We found that introns inhibited the expression of their genes in the *RPL22* pair, with the *RPL22B* intron conferring a much stronger effect. While the WT *RPL22A*/*RPL22B* mRNA ratio was 93/7, the *rpl22a*Δ*i*/*RPL22B* and *RPL22A*/*rpl22b*Δ*i* ratios were >99/<1 and 60/40, respectively. The intron in *RPL2A* stimulated the expression of its own gene, but the removal of the other introns had little effect on expression of the corresponding gene pair. Rpl22 protein abundances corresponded to changes in mRNAs.

Using splicing reporters containing endogenous intron sequences, we demonstrated that these effects were due to the inhibition of splicing by Rpl22 proteins but not by their RNA-binding mutant versions. Indeed, only WT Rpl22A/Rpl22B proteins (but not the mutants) interacted in a yeast three-hybrid system with an *RPL22B* intronic region between bp 165 and 236. Transcriptome analysis showed that both the total level of Rpl22 and the A/B ratio were important for maintaining the WT phenotype. The data presented here support the contention that the Rpl22B protein has a paralog-specific role.

The *RPL22* singleton of *Kluyveromyces lactis*, which did not undergo whole genome duplication, also responded to Rpl22-mediated inhibition in *K*. *lactis* cells. Vice versa, the overproduction of the *K*. *lactis* protein reduced the expression of *RPL22A/B* in *S*. *cerevisiae*. The extraribosomal function of of the *K*. *lactis* Rpl22 suggests that the loop regulating *RPL22* paralogs of *S*. *cerevisiae* evolved from autoregulation.

## Introduction

Ribosome biogenesis absorbs a substantial portion of yeast cell resources [1]. It requires 78 ribosomal proteins (RPs), approx. 200 ribosomal protein assembly factors, and ~75 small nucleolar RNA genes [2]. All of this production needs to be precisely balanced and, perhaps more importantly for regulatory requirements, must be capable of coping with the rapid changes in ribosome numbers. Cells must be able to react to the availability of nutrients and to various stressors by speeding up or stopping growth [3]. In a fast growing cell, ribosomes must be assembled at a rate of 2000 per minute to a degree of high precision and efficiency [1,4], while leaving no unused components or stalled assembly intermediates that would otherwise hamper fitness or even endanger the cell [5,6].

Ribosomal proteins in *S*. *cerevisiae* are encoded by 137 genes, among which are 59 pairs of functional paralogs [7]. These paralogs originated during the events leading to whole genome duplication (WGD) in the ancestor of *Saccharomyces* before 100 My and have been retained to a considerable degree, representing ~11% of the surviving paralog pairs [8–10]. It is believed that pairs of ohnologs (paralogs that originated during WGD) brought about a higher level of regulatory complexity, increasing the spectrum of responses to varying nutrient conditions [11,12]. A control system originally regulating the expression of one ancestral gene can become intergenic [13]. *S*. *cerevisiae* is an intron-poor species that has lost most of its introns (only ~280 remain) [14,15]. Of these, 104 are RPG introns; therefore, given the high expression levels of RPGs [16,17], 90% of mRNA splicing is devoted to producing RPG mRNAs [1].

Although RPG paralogs mostly code for proteins of an identical or highly similar amino acid composition (35 pairs differ by less than 2 amino acids [18]), they differ markedly in their 5’- and 3’-UTR sequences and introns [19]. These differences are species/strain-specific and, at least in yeast, have been shown as subject to selection pressure [20]. Apparently, they were fixed in populations as a result of adapting to specific growth conditions and genetic backgrounds [20,21]. Some RPG pairs differ in their ORFs and their products have distinct localization patterns. These factors can affect specific aspects of cell physiology. For example, it has been reported that translation of *ASH1* mRNA requires a specific subset of ribosomal protein paralogs, including Rpl22A [22]. Shifts in the relative concentrations of paralogs can lead to subpopulations of functionally different ribosomes [23]. “Specialized” ribosomes can operate in distinct subpools of mRNA substrates or in specific compartments [22,24–26]. Recently, quantitative mass spectrometry of translationally active ribosomes from mouse embryonic stem cells confirmed ribosomal heterogeneity with respect to several RPs as well as their association with specific subsets of mRNAs [27].

RPG expression in *S*. *cerevisiae* is tightly coordinated with rRNA synthesis [28,29] and predominantly regulated at the levels of transcription [3,30] and splicing [31]. Systematic deletions of introns from RP genes indicate that introns affect cell fitness, rRNA processing, and the production of other paralogs in the case of duplicated RPGs [23]. Intron-containing RPGs have been shown to respond to various types of environmental stresses [21,23,31]. The mechanisms implicated in splicing-mediated regulation include the interaction of the ribosomal protein with its own (or paralogous) transcript through motifs related to rRNA structures bound by RP [32]. The ability of intramolecular pre-mRNA structures to influence splicing reactions was first established more than 30 years ago [33,34], but detailed understanding of the structural properties of introns has only recently begun to emerge [35]. Complementary sequences can base-pair over shorter or longer distances, looping out exons [36], occluding or weakening splice sites [37], or bringing splice sites into proximity [35,38,39]. In more complex scenarios, splicing signals are only modulated by the structural context, which can impact/block spliceosome assembly at later stages. One of the most extensively studied examples of intron-mediated regulation is *RPL30*. The Rpl30 protein inhibits its own synthesis by blocking both the splicing and translation of the *RPL30* transcript [40–42]. Rpl30 binds an internal loop in the vicinity of 5’ss and affects U2-mediated recognition of BP, perhaps through its effects on the U1 snRNP conformation or the structure of the transcript [43]. Other examples of splicing regulation include Rps14, which binds to *RPS14B* (but not *RPS14A*) pre-mRNA and represses its splicing [44], as well as Rps9 and Rpl22, which inhibit the splicing of *RPS9A* and *RPL22B* pre-mRNA, respectively [13,45]. These inter-paralog relationships seem to have evolved independently and in some cases several times in different taxa [13].

A high proportion of RPG pairs is unique to post-WGD yeast. However, in the cases of some RPGs, paralogs have occurred throughout evolution. The Rpl22 pair is one of the better-characterized examples of such a tendency. The available evidence documents its existence in yeast, zebrafish, *Drosophila*, mice, and humans [23,46–48]. Rpl22 and Rpl22-Like1 play extraribosomal roles in modulating the splicing of Smad2 pre-mRNA during zebrafish morphogenesis. They both bind Smad2 pre-mRNA but act antagonistically; their balance is important for correct splicing of exon 9 [49]. Rpl22 is required for T-cell differentiation in mice and for the emergence of hematopoietic stem cells in zebrafish [46]. Mouse Rpl22 binds to Rpl22l1 mRNA and regulates its expression [48]. During Epstein-Barr virus infection, human Rpl22 is sequestered by EBER-1 viral RNA, a condition which enhances the replicative potential of Burkitt lymphoma cells [50]. Human *RPL22* has also been suggested as playing a role in binding to telomerase RNA [51]. Several RPs, including Rpl22, have been implicated in p53 activation upon stress disturbing ribosome biogenesis [52,53]. Rpl22 has been recently shown to directly bind and block MDM2 [53], which is intriguing, given the occurrence of inactivating mutations of *RPL22* in various cancers [53,54]. In yeast, *RPL22A* deletion increases replicative lifespan [18] and affects sensitivity to oxidative stress [55,56] and acetic acid [57]. Furthermore, Rpl22 has been implicated in translation regulation of meiotic inducer *IME1*. The atypical 5’-UTR of the *IME1* transcript stalls its translation in *rpl22*Δ cells [58]. Recently, Gabunilas and Chanfreau reported that *RPL22* paralogs are regulated in an intergenic fashion through their introns. This regulation was mediated by Rpl22 proteins and required a structured element that did not show direct sequence homology with the rRNA binding motif to which Rpl22 binds within the ribosome [59].

In our study, we set out to analyze the roles of introns in intergenic relationships observed previously in *S*. *cerevisiae* [23]. Of the 7 paralog pairs examined, we found 1 intragenic and 1 intergenic intron-dependent effect in *RPL2A* and *RPL22A/B*, respectively. In this paper, we summarize our findings on the *RPL22* pair and independently demonstrate–using proteomic analysis of Rpl22 levels, three hybrid system testing, and assays with RNA-binding mutants–that intergenic regulation is mediated by Rpl22 protein-binding and is asymmetric with respect to introns. A and B introns are capable of inhibiting mRNA production of their own genes in an Rpl22-dependent way to 50% and 8% of WT levels, respectively. Importantly, we show that *Kluyveromyces lactis RPL22*, which is not a duplicated gene, is also subject to intron- and Rpl22 protein-mediated inhibition in *K*. *lactis* cells. Because Rpl22 can potentially bind to other non-ribosomal targets as well as its own pre-mRNA [49], we mapped the effects of *RPL22A*/*RPL22B* gene and intron deletions on the transcriptome level. Our data suggest that Rpl22B assumes a specific role which cannot be complemented by Rpl22A.

## Methods

### Strains, growth conditions, transformation

The strains used in this study were derived from BY4741 and BY4742 standard laboratory strains (the list is provided in S1 Table). For gene expression analysis, yeast were grown for at least eight generations in a rich medium (YPD supplemented with adenine, YPAD) after transferring from a pre-culture until they reached the mid-exponential phase. For overexpression and primer extension analysis, cells were grown in a synthetic medium without uracil and/or histidine for two generations. Yeast transformation was performed as described [60].

### Intron deletions

The *delitto perfetto* method was used to delete introns from *RPL22* genes in the BY4741 and BY4742 strains [61]. In the first step, we amplified the *URA3* gene from the pRS316 plasmid with primer pairs KA70 + KA71 and KA78 + KA79 and inserted it into the *RPL22A* and *RPL22B* intron, respectively. In the second step, cells were transformed with integrative recombinant oligonucleotides (IROs), KA72 + KA73 for *RPL22A* and KA80 + KA81 for *RPL22B*, whose integration led to the loss of the *URA3* gene together with the intron sequence. These cells were selected using 5-fluoroorotic acid (5-FOA). To delete the hairpin from the *RPL22A* intron, we used IROs KA74 + KA75. *KlRPL22* intron was removed from JLQ36 strain using the procedure described below (simplified). *URA3* cassette was amplified with primers JL479 and JL480 from pJet1-*RPL22A*-*RPL22Bi*-*URA3*. URA3^+^ transformants were left overnight on YPAD plates before selection on 5-FOA. Successful intron/hairpin deletions were confirmed by PCR and sequencing. The list of all oligonucleotides and plasmids used in this study is provided in S2 and S3 Tables, respectively.

### Intron and gene replacement

Intron and gene swaps were performed using an approach similar to the method used by Längle-Rouault [62]. Briefly, we prepared DNA cassettes containing the sequence to be inserted, flanked by 45–50 bp arms complementary to the immediate surroundings of the destination site. The 3’ flanking sequence was included twice as a direct repeat separated by the *URA3* selection marker. While *URA3* was used to select transformed cells, it was also prone to pop out via homologous recombination between the two copies of the 3’ flanking sequence. These cells were then selected on a medium containing 5-FOA. The genetic manipulation was confirmed by sequencing. S4 Table summarizes the oligonucleotides used and the mode of creation for particular constructs. Two of the replacement cassettes were constructed in the pJet1 plasmid. PCR fragments produced with primers F1 + R1 (5’ part containing the sequence to be inserted + flanking nucleotides) and F2 + R2 (3’ part–the *URA3* marker and the second copy of the 3’ flanking sequence) were first inserted individually into the pJet1 plasmid using the CloneJet kit (Thermo Fisher Scientific). The respective F1-R1 fragments were then excised by digestion with BamHI and BglII and ligated into the corresponding F2-R2-bearing constructs digested with BglII. The other cassettes were assembled by PCR with primers F1 and R2 using an overlap of 24 bp between the parts.

### Plasmid construction

To generate the *RPL22* overexpression constructs, the respective *RPL22* ORFs were PCR-amplified from genomic DNA from intronless strains (KAY61 strain and primers FN12 + FN13 for *RPL22A*; KAY67 strain and primers FN14 + FN15 for *RPL22B*), digested with NcoI/BamHI, and cloned into plasmid pVTU260 digested with the same enzymes. *KlRPL22* was amplified from *Kluyveromyces lactis* IFO1267 cDNA using primers FN34 and FN35 and cloned in the same manner. For experiments using *K*. *lactis*, expression cassettes containing *RPL22A*, *RPL22B*, their mutated versions, and *KlRPL22* were released from pVTU260 by PaeI digestion and inserted into the pCXs22 plasmid [63].

Mutations in the Rpl22 RNA binding site [64] were designed to change lysines in the protein sequence 73-GKYLKYLTKKYLKKNQL-89 to glutamates (73-GEYLEYLTEEYLEENQL-89) by changing the lysine codons to GAA. DNA sequences coding for the Rpl22 RNA-binding mutants flanked with NcoI and BamHI restriction sites were synthesized by GeneArt (Thermo Fisher Scientific) and used to replace WT *RPL22* ORFs in the pVTU260 plasmid with the corresponding restriction enzymes.

To prepare the splicing reporters, we replaced the BamHI/EcoRI *COF1* insert in the pOG71 plasmid [39] with BamHI and EcoRI-digested products of PCR amplification with primers MO09 + MO10 for the *RPL22A* intron and primers MO11 + MO12 for the *RPL22B* intron using BY4741 genomic DNA as a template. These manipulations resulted in plasmids p423GPD-*RPL22A*-*CUP1* and p423GPD-*RPL22B*-*CUP1*, which were verified by restriction analysis and sequencing.

Vectors p3HR2 and pACT2 used in the yeast three-hybrid assays were donated by the Wickens Laboratory. To create hybrid proteins, intronless versions of *RPL22A*, *RPL22B*, and their RNA-binding mutants were re-cloned from the pVTU260-based expression vectors into the pACT2 vector using the NcoI and BamHI restriction sites. The intronic sequences were PCR-amplified from genomic DNA (strain BY4741) and cloned into the SphI site of vector p3HR2.

### RNA isolation, reverse transcription and real-time PCR

RNA from cells grown to the mid-exponential phase in the YPAD medium was isolated using the MasterPure Yeast RNA Purification Kit (Epicentre Biotechnologies), including the DNase I treatment step, essentially according to the manufacturer’s instructions. Two micrograms of RNA were converted to cDNA using RevertAid reverse transcriptase (Thermo Fisher Scientific) with random hexamer primers unless indicated otherwise. Real time-qPCR reactions were performed using the MESA GREEN qPCR MasterMix Plus for SYBR Assay No ROX (Eurogentec). All reactions were run in triplicate using the LightCycler 480 II (Roche). Relative pre-mRNA and mRNA quantities were calculated using the ΔΔCt method [65]. For intron and gene replacement experiments, primers were designed in order to distinguish between the various forms of *RPL22* transcripts present in the cells. Specificities of the primer pairs used are listed in S5 Table. All primer pairs were tested for lack of (i) unspecific amplification under standard qPCR conditions and (ii) amplification of other related templates mentioned in S5 Table. The contribution of the second copy of *RPL22B* in the strain where the intron and exon 2 of *RPL22A* were replaced with the corresponding part of *RPL22B* was calculated as the difference between the relative abundances of total *RPL22B* mRNA (primers KA50 + JL385) and *RPL22B* mRNA transcribed from the *RPL22B* locus (JL360 + JL385).

The statistical analysis was performed on ΔC_t_ data using the t-test with correction for multiple testing. Statistical tests were conducted with the "R" statistical package version 3.2.3 (www.r-project.org/) using the t.test() function with parameters “paired = FALSE, alternative = 'two.sided' “. The function p.adjust() with Holm correction was used; *P<*0.05 was considered significant.

### Primer extension analysis of relative splicing efficiency

The analysis was performed according to the protocol described in [39]. Briefly, RNA was isolated using the MasterPure Yeast RNA Purification Kit (Epicentre Biotechnologies). Three to five micrograms of RNA were transcribed into cDNA using RevertAid reverse transcriptase (Thermo Fisher Scientific) with the YAC06 and YU14 [66] primers labeled using γ^32^P-ATP and the T4 polynucleotide kinase (Promega). cDNA molecules were then separated on 8% polyacrylamide gel with 7 M urea together with a ΦX174 HinfI DNA labeled marker. Radioactive signals were captured on Imaging Screen-K (BioRad) and detected using the Typhoon FLA imager (GE Healthcare Life Sciences).

### Proteomic analysis

For mass spectrometry (MS) analysis, cells were grown in 50 ml of YPAD to the mid-exponential phase, collected by centrifugation (1000 g, 3 min), washed with 1 volume of water, frozen in liquid nitrogen, and stored at -80°C. Cells were disintegrated by 5 rounds of beating (20 s, speed 4, 5 min cooling between rounds) with glass beads in the FastPrep-24 instrument (MP Biomedicals) in 450 μl of 100 mM triethylammonium bicarbonate buffer with 1% sodium deoxycholate. Cell debris was removed from the lysate by centrifugation at 1000 g for 1 min at 4°C, followed by centrifugation at 2000 g for 5 min at 4°C; the lysate was frozen and stored at -80°C. Peptides were generated by trypsin digestion, analyzed on Thermo Orbitrap Fusion (Q-OT-qIT, Thermo), and quantified using MaxQuant software (version 1.5.3.8) [67] in our core facility.

### Yeast three-hybrid protein-RNA interaction testing

Interactions of Rpl22 proteins with their RNA ligands were assayed in the YBZ1 yeast strain as described in [68]. Transformants containing plasmids expressing the respective hybrid protein and target RNA were assayed for expression of the *HIS3* reporter gene by testing the ability of 10-fold serial dilutions of cell cultures to grow on selective media. To distinguish between interactions of different strength, activity of the *HIS3* reporter was assessed in the presence of a competitive inhibitor, 3-aminotriazole.

### RNA structure modeling

For RNA structure modeling, RNAFold [69] was used with default settings, except for the temperature being set to 30°C.

### Transcriptome profiling

Cells grown in the exponential phase for at least eight generations in YPAD medium at 30°C were harvested by centrifugation (1000 g, 3 min, room temperature); cell pellets were stored at -80°C. Cells were disrupted using glass beads in the FastPrep-24 instrument (MP Biomedicals) and total RNA was obtained using the acidic phenol:chloroform extraction method as described previously [70].

RNA was further purified with the MasterPure Yeast RNA Purification Kit (Epicentre Biotechnologies), starting with the DNase I treatment step. Strand-specific paired-end RNA-Seq libraries were constructed from poly(A)-enriched RNA samples by BGI Genomics using their standard procedure. RNA-Seq libraries were sequenced (100 nt, paired-end) by BGI Genomics using the Illumina HiSeq platform. Raw reads were filtered after sequencing, including the removal of adapter sequences, contamination, and low-quality reads. Three biological replicates were analyzed for each strain. The sequencing data are available in the ArrayExpress database (www.ebi.ac.uk/arrayexpress) under accession number E-MTAB-5275.

The quality of the sequencing reads was checked with fastQC 0.11.4 (www.bioinformatics.babraham.ac.uk/projects/fastqc). Reads were aligned to the *S*. *cerevisiae* genome r64 using HISAT 2.0.3-beta [71]. Non-uniquely mapping reads (MAPQ < 10) were filtered out using samtools 1.3.1 [72]. Analysis of differential gene expression was performed using R 3.2.3 (www.r-project.org) and the Bioconductor package DESeq2 [73,74] at a 5% false discovery rate. Significance of overlaps between lists of DEGs was determined by Fisher’s exact test (function fisher.test()); antagonistic regulation of *rpl22b*Δ DEGs in the *rpl22a*Δ strain was tested by one-sided Wilcoxon signed rank test (function wilcox.test()). Splicing efficiency for each intron was calculated as described previously [75]. Briefly, transreads (reads spanning exon-exon junctions) were extracted using regtools 0.2.0 (https://regtools.readthedocs.io). Intron 5’ end base coverage was determined using bedtools 2.25.0 [76]. Splicing efficiency was then calculated as the ratio of transreads (mRNA) to intron-end reads (pre-mRNA). Enriched GO categories were searched for using GO Term Finder 0.83 (http://www.yeastgenome.org/cgi-bin/GO/goTermFinder.pl).

## Results

### Introns in some, but not all, RPGs affect their gene expression under standard cultivation conditions

**Fig 1:**
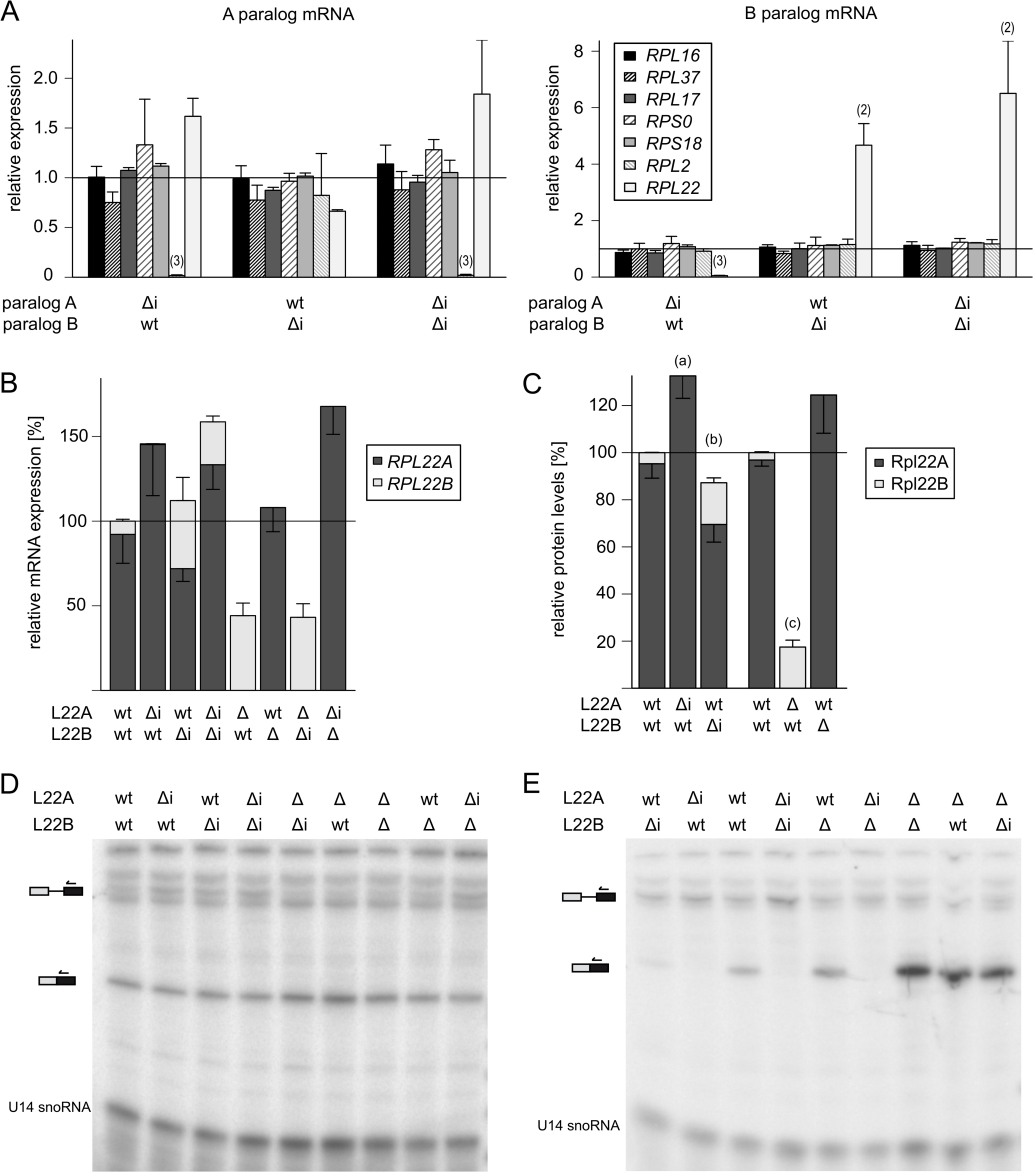
Introns mediate intergenic regulation of *RPL22* paralogs. (A) Impact of intron deletions on the expression of duplicated ribosomal protein genes. Transcript levels of *RPL16*, *RPL17*, *RPL37*, *RPS0* and *RPS18* paralogs are refractory to intron deletions. In contrast, *RPL22* and *RPL2A* show intergenic and intragenic intron dependency, respectively. Plots show mean fold changes of “A” (a) and “B” (b) paralog mRNA in mutants with intron deletion (Δi) in “A”, “B” or both paralogs as determined by RT-qPCR relative to WT. Data were normalized to *SPT1*5 expression and to the RNA level in WT cells. Error bars represent s.d. from two (*RPL17*, *RPS0*, *RPS18*) or three (*RPL2*, *RPL16*, *RPL37*) biological replicates. The statistical significance of the difference between WT strain and a strain bearing intron deletion is indicated as (2) for *P*≤0.01 and (3) for *P*≤0.001 based on the t-test with Holm correction for multiple testing (see Methods). Pre-cultures of colonies from freshly dissected spores were diluted to such a low OD so that they could undergo 10 generations before they were harvested at mid-exponential phase. In independent experiments, intron deletion mutants of *RPL22*, *RPL16* and *RPL37* were grown in YPAD or synthetic medium for 2 or 10 generations, respectively. The two cultivations gave essentially the same results as above (data not shown). (B) *RPL22* mRNAs are negatively regulated by their introns and respond in an intergenic way to the manipulations of their paralogous counterparts. The plot shows mRNA levels of *RPL22* paralogs normalized to WT. The *RPL22A* to *RPL22B* ratio was calculated from the same data as shown in S1 Fig. Mean values ± s.d. from at least three biological replicates are shown (for details and statistical analysis, see S1 Fig). (C) Rpl22 protein levels reflect the changes in corresponding mRNAs. Relative protein levels were assessed based on mass spectra intensities measured from whole cell lysates. Mean values ± s.d. from 3 biological replicates are shown. *P* values were obtained for comparisons of log-normalized protein intensities (Label Free Quantification Algorithm; MaxQuant, see Methods) between WT strain and a mutant strain using the t-test with Holm correction for multiple testing. Significant differences are indicated as (a) *P* = 0.0182 for Rpl22A (Rpl22B intensity was below detection limit), (b) *P* = 0.0297/ 0.0026 for Rpl22A/ B, and (c) *P* = 0.0006 for Rpl22B. (D,E) Splicing efficiency analysis of *RPL22A* and *RPL22B* introns, respectively, in *RPL22*-manipulated strains. Gels show radioactively labeled primer extension products from cells expressing *RPL22A-CUP1* (D) and *RPL22B-CUP1* (E) reporter substrates. U14 snoRNA was used as a loading control. Each gel is representative of at least three independent experiments.

The roles of RPG paralogs in ribosome biogenesis and translation, both in unicellular eukaryotes and in Metazoa, remain an important issue [20]. We tested 7 pairs of paralogous gene pairs–*RPL22*, *RPL16*, *RPL37*, *RPL17*, *RPL2*, *RPS0*, and *RPS18 –*previously reported to be regulated in an intergenic and intron-dependent way [23]. From diploid strains containing heterozygous intron deletions in each paralog, we prepared haploids lacking the intron in either the A paralog, B paralog, or in both and then assayed RPG mRNA abundance (Fig 1A). Intron deletions in all but two of the tested candidate pairs had little impact on expression levels of the manipulated gene or its paralog. Intron deletion in *RPL2A* led to a very strong inhibition of its own expression, which points to the existence of an intragenic regulatory loop, while intron deletions in *RPL22A/B* showed intergenic effects. Phenotypes of the intron-manipulated *RPL22* strains were reproducible across cultivation conditions (YPAD versus synthetic media), irrespective of the strain background (BY4741/BY4742 versus JPY10I) or the number of generations between the inoculation and the collection of cells for analysis. Further analysis of additional environmental stresses and metabolic states may clarify the differences between our study and the previous results of Parenteau et al. [23]. Notably, there are also discrepancies between studies with regard to changes of intron deletions in the *RPS9* and *RPS14* paralogs [13,44,23]. In summary, while some paralog pairs display intron-dependent intergenic relationships, the roles of introns in other pairs may be less obvious and should not be simply inferred.

### Introns mediate asymmetric intergenic regulation of the *RPL22A* and *RPL22B* paralogs by Rpl22 proteins

Because we were able to observe an intergenic relationship only with the *RPL22A/B* gene pair, we next concentrated on it. We prepared intronless versions of *RPL22A* and *RPL22B* in the BY4741 and BY4742 genetic backgrounds, compatible with yeast deletion collection [77]. As shown in Fig 1 and S1 Fig, the effects of *RPL22* intron deletions in the strains derived from BY4741 and BY4742 are in agreement with the results obtained using strains constructed by J. Parenteau [23]. The ratio of *RPL22A* to *RPL22B* mRNA abundance was 93 to 7 in WT (Fig 1B). Intron deletion from *RPL22A* (designated *rpl22a*Δ*i*) led to a ~1.6-fold increase in *RPL22A* mRNA, accompanied by a dramatic (~19-fold) decrease of *RPL22B* mRNA. Removal of the intron from *RPL22B* (*rpl22b*Δ*i*) enhanced the abundance of its own mRNA 5.4-fold, while *RPL22A* expression decreased 1.3-fold.

The expression of *RPL22A* did not differ considerably from the WT level when the *RPL22B* gene was deleted, but it did differ considerably when the *RPL22A* intron was also absent. On the other hand, removing *RPL22A* increased *RPL22B* mRNA to a similar abundance as in *rpl22b*Δ*i* (40% of total *RPL22* mRNA in WT). Still, the level of total *RPL22* mRNA was lower in *rpl22a*Δ than in WT, which suggests that these cells are unable to attain the normal level of *RPL22* expression by transcription from the *RPL22B* locus alone. We also tested the levels of pre-mRNA in all the above strains (S1 Fig). mRNA downregulation of *RPL22A* and *RPL22B* in *rpl22b*Δ*i* and *rpl22a*Δ*i*, respectively, was not accompanied by dramatic increases in corresponding pre-mRNAs. In contrast, upregulation of *RPL22B* mRNA in *rpl22a*Δ, which apparently represents the maximum expression attainable from that locus, was accompanied by a drop in pre-mRNA levels.

To test whether mRNA levels would also be reflected in protein abundance, we analyzed the relative amounts of Rpl22A and Rpl22B proteins in strains with different *RPL22* alleles using mass spectrometry. The proteomic data corresponded to those for mRNA levels (compare Fig 1B and 1C). Rpl22A was the major Rpl22 protein in cells. Its abundance increased in cells where its gene did not contain an intron as well as in the *rpl22b*Δ strain where Rpl22B was missing. On the other hand, Rpl22B accounted only for 4% of the total Rpl22 level in the WT strain. However, when its intron-mediated regulation was abrogated, the Rpl22B level rose to ~17.5%.

Both *RPL22* genes contain long introns (389 nt in *RPL22A*, 321 nt in *RPL22B*). The distance between the branch-point and 3’ splice-site is longer in *RPL22A* (56 nt) than in *RPL22B* (17 nt). The two introns also differ in their donor site sequences, which both deviate from the GTATGT consensus: GTATGA in *RPL22A* and GTACGT in *RPL22B*. Interestingly, there is potential for a stable secondary structure (hairpin) to be formed between the branch-point and 3’ splice site of the *RPL22A* intron (S2 Fig). However, when we prepared a strain from which the hairpin was removed (*rpl22a*Δ*H*), the levels of *RPL22* mRNAs and pre-mRNAs in that strain did not differ from WT (S1 Fig).

The asymmetric behavior of these introns led us to analyze the splicing efficiency of *RPL22*-derived splicing reporter constructs in strains with manipulated *RPL22* genes (Fig 1D and 1E). In these reporters, the strong constitutive GPD promoter drove transcription of exon 1 (including a part of 5’-UTR) followed by the intron and a part of exon 2 of either *RPL22A* or *RPL22B* fused to the *CUP1* gene. Using a primer extension, we observed high-efficiency *RPL22B* reporter splicing in strains from which the *RPL22A* paralog was absent (Fig 1E), which supports the hypothesis that Rpl22A stalls the expression of *RPL22B* by blocking the splicing step. This inhibition probably occurs during splice-site recognition or during the early steps of spliceosome assembly, as we did not observe any lariat-intermediate accumulation. Indeed, splicing of the *RPL22B* reporter was inhibited in strains with intron deletion in *RPL22A* (i.e., with increased Rpl22A protein levels), whereas it was intermediate in WT. In contrast to *RPL22B*, the relative changes of *RPL22A* reporter mRNA accumulation were much less pronounced (Fig 1D). To summarize, we documented that *RPL22* introns inhibit expression of their genes, albeit to a different extent. Rpl22 protein levels corresponded with mRNA levels.

### *RPL22* pre-mRNAs are controlled by the nonsense-mediated decay pathway

**Fig 2:**
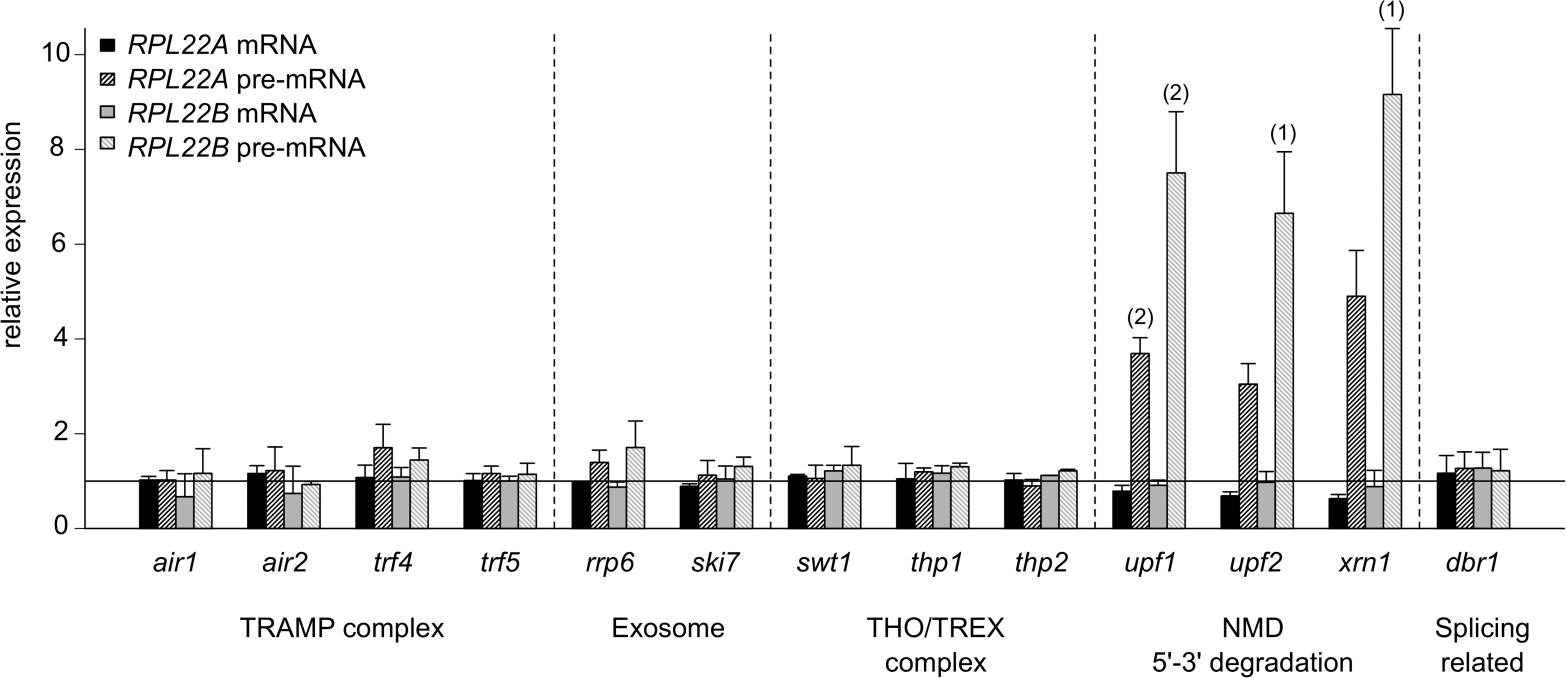
*RPL22* pre-mRNAs are targeted by the nonsense-mediated decay pathway. *RPL22A* and *RPL22B* expression was assessed by RT-qPCR (reverse transcription followed by quantitative PCR) in strains with deletion in genes representing various pathways of RNA metabolism. cDNA was prepared using random hexamers and oligo(dT) 18-mers. Values for each amplicon were normalized to WT levels and are represented by a horizontal line. The means of biological triplicates, quadruplicates (*air1*Δ), and duplicates (*ski7*Δ, *swt1*Δ, *thp1*Δ, and *thp2*Δ) are shown. The statistical significance of the difference between WT strain and a mutant strain is indicated as (1) for *P≤*0.05 and (2) for *P≤*0.01 based on the t-test with Holm correction for multiple testing (see Methods).

Regardless of changes in mRNA quantity, *RPL22* pre-mRNA levels were mostly unaffected (S1 Fig). To determine which RNA degradation pathways would be involved in the metabolism of *RPL22* RNA species, we analyzed changes in mRNA and pre-mRNA levels of *RPL22A* and *RPL22B* in strains with deletions of genes involved in several RNA degradation pathways (Fig 2). Members of the TRAMP complex (*air1*Δ, *air2*Δ, *trf4*Δ, *trf5*Δ), exosome complex (*rrp6*Δ, *ski7*Δ, *hbs1*Δ), and THO/TREX complex (*swt1*Δ, *thp1*Δ, *thp2*Δ) did not affect the levels of *RPL22* RNA species. On the other hand, deletions of cytoplasmic nonsense-mediated decay (NMD) pathway members (*upf1*Δ, *upf2*Δ, and *upf3*Δ) and cytoplasmic 5’-3’ exonuclease *xrn1*Δ increased pre-mRNA levels of *RPL22A* ~4 fold and *RPL22B* ~8 fold.

To obtain more detailed information about *RPL22* RNA metabolism, we analyzed double-mutant strains with a deletion of selected RNA surveillance genes (*air1*Δ, *air2*Δ, *dbr1*Δ, and *upf1*Δ) together with *RPL22* intron manipulations (S3 Fig). Impairment of the pathways in *air1*Δ, *air2*Δ, and *dbr1*Δ did not change the expression profiles of *RPL22* mRNAs and pre-mRNAs beyond the effect of intron manipulations. Importantly, *RPL22B* pre-mRNA was 8 times more abundant in *rpl22a*Δ*i upf1*Δ than in WT cells, even though *RPL22B* mRNA levels remained very low. This further supports the hypothesis that *RPL22B* pre-mRNA splicing is blocked by elevated levels of Rpl22.

We also took advantage of a mutant in splicing factor *PRP45* that causes pre-mRNA accumulation due to impaired spliceosome assembly [78]. We compared the effects of this splicing factor mutation (*prp45*(1–169)) with that of *upf1*Δ, *upf3*Δ, and *xrn1*Δ on pre-mRNA accumulation of *RPL22A/RPL22B*, *ECM33*, and *COF1* transcripts (S4 Fig). Unlike *ECM33*, where *prp45*(1–169) elicited ~6-fold pre-mRNA accumulation (NMD alleles had little effect), *RPL22B* showed the opposite behavior. The *RPL22A* response was intermediate. We suggest that these data reflect the low proportion of splicing events per pre-mRNA molecule generated from *RPL22B* as well as the dependence of *RPL22* metabolism on NMD. In summary, we suggest that both the NMD pathway and 5’-3’ cytoplasmic degradation are responsible for pre-mRNA degradation of both *RPL22A* and *RPL22B* in WT cells.

### Overexpression of Rpl22A/B, but not their mutant versions, downregulates the production of *RPL22* mRNAs in an intron-specific way

**Fig 3:**
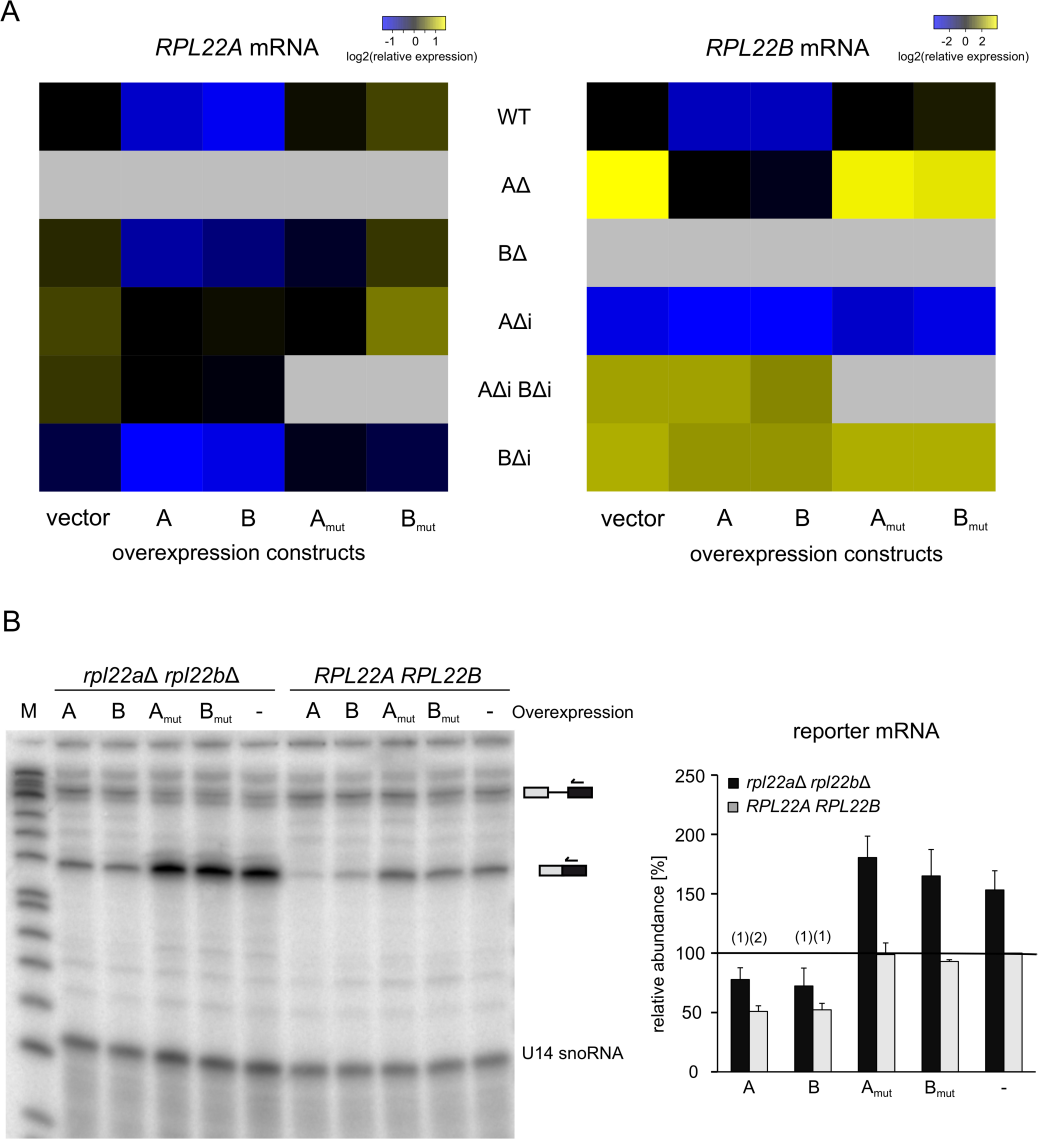
Overproduction of Rpl22A and Rpl22B, but not their mutant forms, downregulates endogenous *RPL22* mRNAs. (A) Heatmaps show endogenous mRNA abundances of *RPL22A* (left panel) and *RPL22B* (right panel) in the indicated genetic backgrounds (arranged in rows) with overexpression constructs (in columns). All values were normalized to WT with an empty vector (top left cell in each heatmap). Leftmost columns (“vector”) reflect only the effect of genetic background. The A/B and Amut/Bmut columns show the effect of overexpression of either WT or RNA binding mutants of Rpl22A or Rpl22B. The “AΔi”, “BΔi”, and “AΔi BΔi” symbols denote strains lacking introns in one or both *RPL22* paralogs. Gray fields indicate that data were not determined. For details and statistical analysis see S6 Table. (B) Primer extension analysis of the relative splicing efficiency of a reporter derived from *RPL22B* (left panel). The effect of overproducing Rpl22A, Rpl22B, and their RNA-binding mutants was compared between a strain lacking endogenous *RPL22* genes and WT. The right panel shows the relative abundance of a spliced reporter mRNA signal normalized to the U14 snoRNA loading control and to the level of mRNA in WT cells with an empty vector. M is the DNA size marker. Error bars represent s.d. from 3 biological replicates. The statistical significance of the difference between empty vector strain and an overexpression strain is indicated as (1) for *P≤*0.05 and (2) for *P≤*0.01 based on the t-test with Holm correction for multiple testing (see Methods).

We attempted to manipulate *RPL22* mRNA levels by expressing the cDNA of each paralog from a multicopy plasmid under the control of the strong *ADH1* promoter. Increased levels of Rpl22A repressed *RPL22B* and *RPL22A* to approx. 10% and 50% of empty vector control, respectively. Rpl22B showed similar efficacies (Fig 3A, S6 Table). Intronless variants of *RPL22A/RPL22B* did not respond to the inhibitory effects of Rpl22A/Rpl22B overproduction, which confirms the intron requirement. To determine whether Rpl22 protein interaction with its primary transcript would be necessary for regulation, we used the same expression system to produce Rpl22 proteins mutated in their putative RNA-binding domain according to a previous study of human *RPL22* [64]. Production of these modified proteins (see S5 Fig) proved inconsequential to endogenous *RPL22* mRNA levels (Fig 3).

We confirmed the capacity of Rpl22 proteins to inhibit splicing using primer extension analysis in *rpl22a*Δ-*rpl22b*Δ double-mutant and WT strains. The strains carried the *RPL22B* splicing reporter and the expression plasmid with or without *RPL22A/RPL22B* cDNA. We observed high-efficiency splicing of the *RPL22B* reporter transcript in the *rpl22a*Δ-*rpl22b*Δ strain, which was strongly blocked by the ectopic production of either Rpl22A or Rpl22B, but not of their RNA-binding mutants (Fig 3B). The same effect was also observed in WT cells, albeit at a smaller amplitude because of the partial splicing block mediated by endogenous Rpl22. Stability of the expressed proteins was confirmed using Western blots (S5 Fig). These results show that introns exert inhibitory effects on *RPL22A* and *RPL22B* mRNA accumulation and that this effect depends on the level of Rpl22 protein(s).

### Introns swapped between *RPL22* genes maintain their regulatory potential

**Fig 4:**
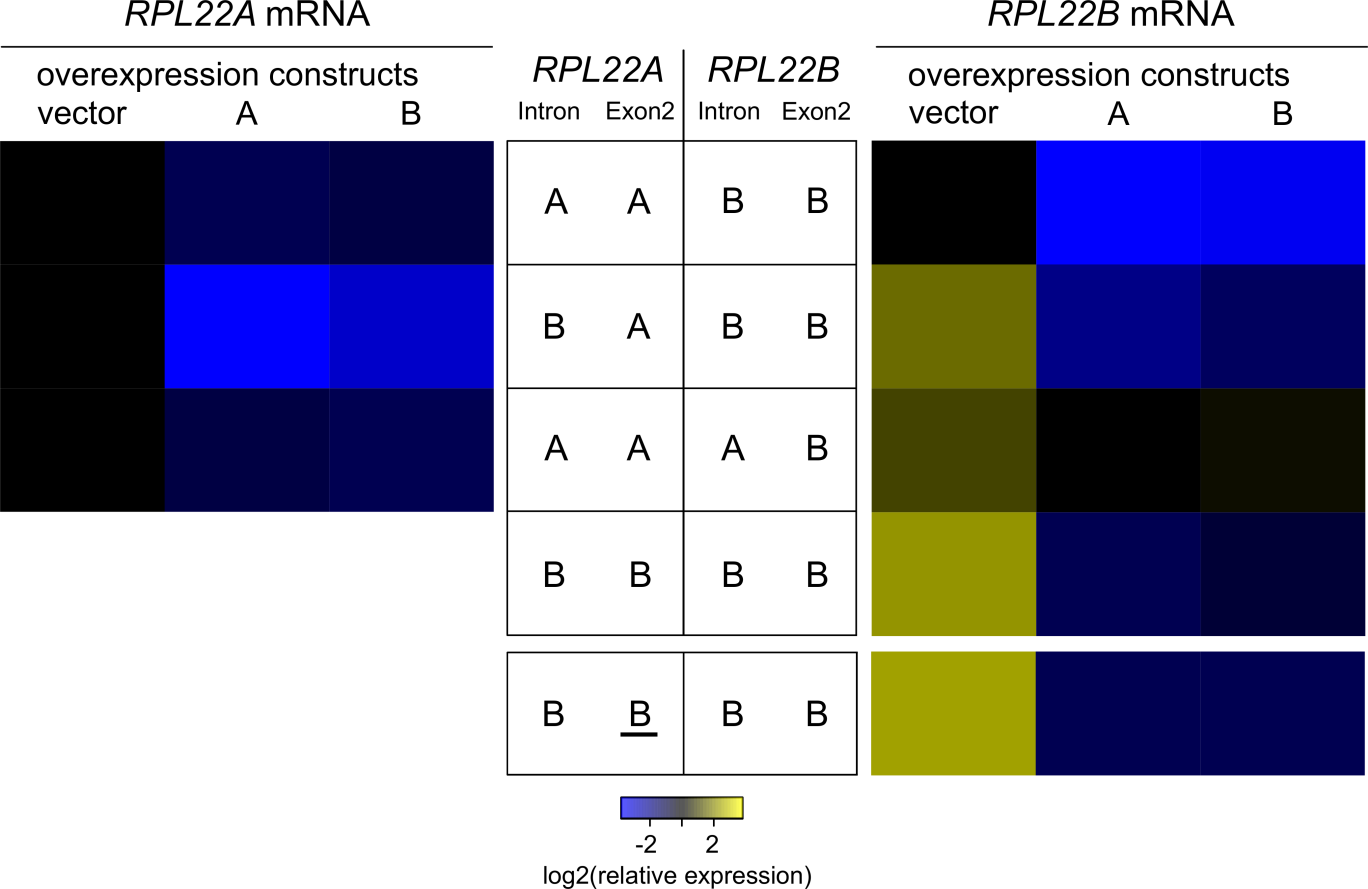
Introns determine the level of sensitivity of *RPL22* expression to downregulation. Heatmaps show endogenous mRNA abundances of *RPL22A* (left panel) and *RPL22B* (right panel) in five genetic backgrounds (arranged in rows) with overexpression constructs (in columns). Configurations of chimeras at the *RPL22A* and *RPL22B* loci are described for each row in the center. All values were normalized to the WT strain bearing an empty vector (top left cell in each heatmap). Leftmost columns (“vector”) reflect only the effect of genetic background, while A/B columns show the effect of overexpression. cDNA was prepared using random hexamers and oligo(dT) 18-mers. The primers used to detect *RPL22B* mRNA were specific for the transcript derived from the endogenous *RPL22B* locus. The detection of the *RPL22B* mRNA transcribed from the A locus (underlined) was accomplished using a specific set of primers (see last row of the right panel). For details and statistical analysis see S7 Table.

To separate the effects of introns from the corresponding coding regions as well as sequences surrounding the locus, we transplanted introns between the *RPL22A* and *RPL22B* genes. Replacing the *RPL22A* intron with its *RPL22B* counterpart led to the decrease of *RPL22A* mRNA and the concomitant derepression of *RPL22B*. Reciprocal transplantation increased *RPL22B* mRNA (Fig 4, S7 Table), which indicates that the *RPL22A* intron confers less stringent inhibition. We then ectopically produced an excess of Rpl22 proteins to test the sensitivity of the chimeric genes to regulation. Overproduction of either Rpl22A or Rpl22B reduced the mRNA abundance of both endogenous *RPL22* genes. There was, however, a significant difference between the sensitivities of the genes containing the *RPL22A* and *RPL22B* introns. Regardless of the locus in which the gene resided and the identity of the coding region surrounding the intron, the presence of the *RPL22A* intron led to mRNA reduction of approx. 50%, while the *RPL22B* intron resulted in a drop of approximately 90% (Fig 4).

We also replaced the *RPL22A* gene (intron + exon 2) with a second copy of *RPL22B*. We took advantage of the fact that *RPL22* exon 1 codes only for 4 amino acids, identical between the paralogs; there is only 1 mismatch among the 12 nucleotides. Therefore, replacing intron + exon 2 (up to the stop codon) effectively swaps the whole coding region. The expression of recombinant *RPL22* was higher than that of the endogenous copy, apparently due to a stronger promoter of *RPL22A*. Irrespective of the expression level, both *RPL22B* genes responded to Rpl22A/Rpl22B overexpression in unison, producing mRNA in similar ratios between empty vector control and overexpression constructs (Fig 4). To reiterate, we confirm that introns determine the sensitivity of *RPL22* expression to Rpl22-mediated inhibition.

### Rpl22 proteins interact with the *RPL22B* intron in a structured region downstream of 5’ss

**Fig 5:**
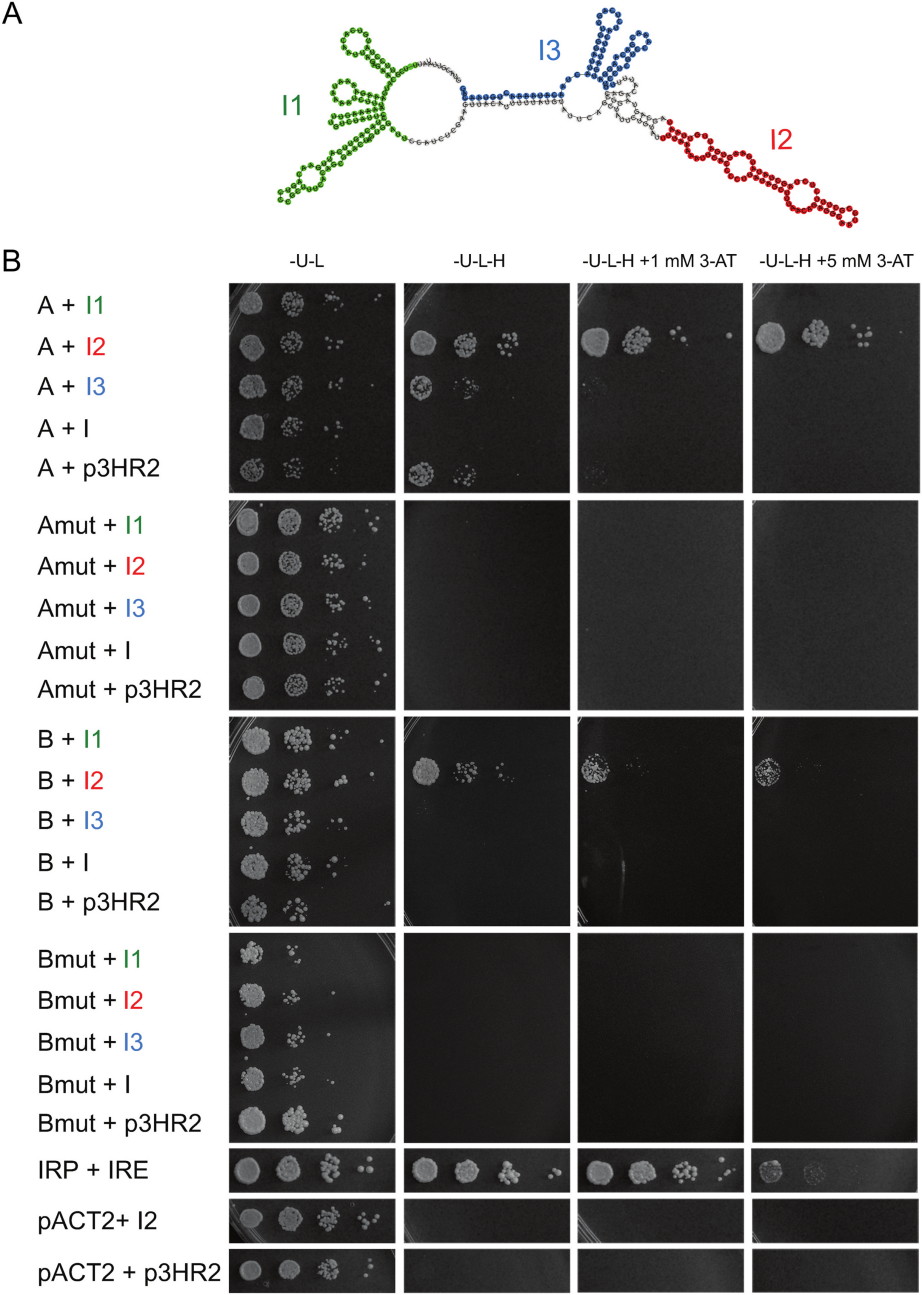
Rpl22A and Rpl22B interact with a specific region of the *RPL22B* intron. (A) RNAfold structure prediction of the *RPL22B* intron (“I”). Regions tested using the three-hybrid system are shown in color. The I1 region (green) represents nucleotides 11 to 123 of the *RPL22B* intron; I2 (red)–nt 165 to 236; I3 (blue)–nt 256 to 321. (B) Intron region 2 (I2) interacts with WT Rpl22A and Rpl22B but not with their RNA-binding mutants using the yeast three-hybrid system. *RPL22A* (“A”) and *RPL22B* (“B”) or their mutated versions (“Amut”, “Bmut”) in combination with different parts of the *RPL22B* intron were assayed for expression of the *HIS3* reporter gene, which is activated in the presence of protein-RNA interaction. 10-fold serial dilutions of cells were spotted on plates with increasing concentrations of 3-aminotriazole (3-AT). “-U”, “-L”, and “-H” denote the lack of uracil, leucine, and histidine in the medium. IRE and IRP served as positive controls. p3HR2 is the empty plasmid for bait RNA expression and pACT2 is the plasmid for expression of the Gal4 activation domain.

Because the interrelationship between *RPL22* paralogs was intron- and protein-dependent, we examined some of the potential Rpl22-intron interactions using the yeast three-hybrid system (3H) [68]. We prepared constructs covering three separate regions of the *RPL22B* intron (see Fig 5) and tested their interaction with Rpl22 proteins as well as their RNA-binding mutants. As shown in Fig 5, we were able to detect the interaction of the “I2” region (nucleotides 165 to 236 of the *RPL22B* intron) with both Rpl22A and Rpl22B. The interaction was comparable in strength to iron regulatory protein 1 (IRP1), with the iron-responsive element (IRE) as the positive control [68]. Rpl22A/Rpl22B RNA-binding mutants, which were successfully produced in the reporter strain (see S6 Fig), did not show any interaction. We also attempted to express the whole *RPL22B* intron in the reporter strain, but no interaction was detected. We assume that because of the stretches of oligo-T, RNA was most likely not produced due to terminating RNA Pol III used in the three-hybrid system [79]. The inhibitory effect of Rpl22 on *RPL22B* pre-mRNA is thus most likely mediated by its binding to a structure within the 72nt region between 5’ss and BP.

### Intron-mediated regulation of *RPL22* is conserved in *Kluyveromyces lactis*

**Fig 6:**
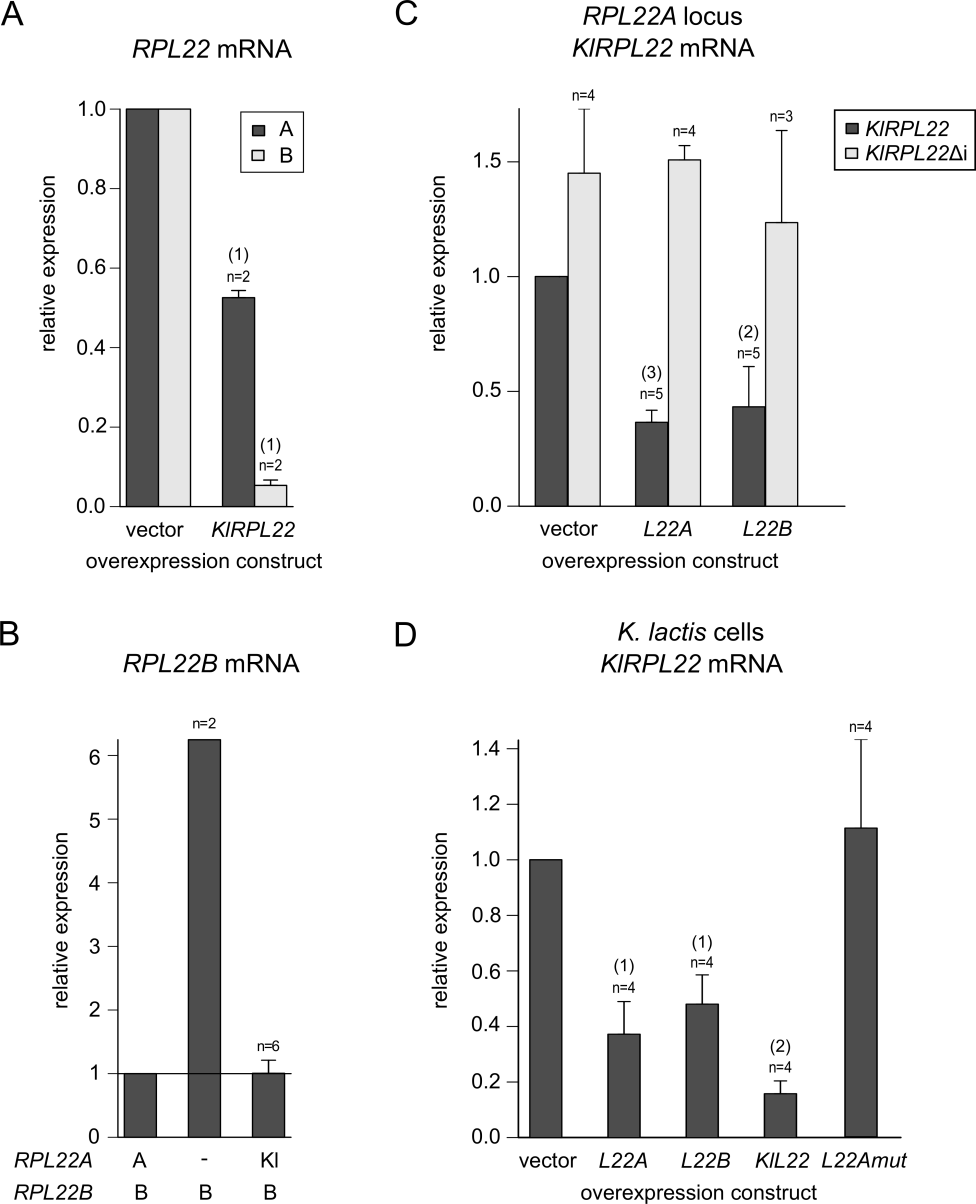
Splicing of *Kluyveromyces lactis RPL22* is subject to Rpl22-mediated inhibition in both *K*. *lactis* and *S*. *cerevisiae*. (A) *KlRPL22* expression in BY4741 cells leads to the downregulation of endogenous *RPL22* mRNAs to an extent similar to that observed for the overexpression of *RPL22A* and *RPL22B* (compare with Fig 3). (B) Negative regulation of *RPL22B* by Rpl22A is maintained when the *RPL22A* intron and exon 2 are replaced with the corresponding part of *KlRPL22*. (C) The abundance of *KlRPL22* mRNA expressed from the *RPL22A* locus as in (B) is decreased by the overproduction of either Rpl22A or Rpl22B. The intronless version of *KIRPL22* in the same locus does not respond to Rpl22A/B overexpression. (D) Endogenous *KlRPL22* mRNA is reduced by the ectopic expression of *KlRPL22* as well as *RPL22A* and *RPL22B* in *K*. *lactis*. cDNA was prepared using random hexamers and oligo(dT) 18-mers. The statistical significance of the difference between empty vector strain and an overexpression strain is indicated as (1) for *P≤*0.05, (2) for *P≤*0.01, and (3) for *P≤*0.001 based on the t-test with Holm correction for multiple testing (see Methods).

*Kluyveromyces lactis* is a member of the “*Saccharomyces* complex” [8,80]. In contrast to the *Saccharomyces sensu stricto* complex taxa, its ancestor did not undergo WGD (see Introduction); *K*. *lactis* has only singleton RPGs in its genome [81]. We therefore wanted to investigate whether Rpl22 would be capable of regulating its splicing in *K*. *lactis*. We ectopically overproduced *K*. *lactis* Rpl22 (further referred to as KlRpl22) in *S*. *cerevisiae*. As shown in Fig 6A, the *K*. *lactis* protein repressed mRNA abundance of endogenous *RPL22A* and *RPL22B* to the same extent as if it were one of the *S*. *cerevisiae* paralogs. To achieve conditions more closely reflecting the physiological situation, we replaced the *RPL22A* gene (intron + exon 2) with the corresponding part of *KlRPL22*. If the *K*. *lactis* protein were unable to complement Rpl22A with regard to its inhibitory capacity, the outcome would be the upregulation of *RPL22B* mRNA. However, in this case *RPL22B* mRNA levels remained at the WT level (Fig 6B). After producing evidence that KlRpl22 is able to regulate *S*. *cerevisiae RPL22* genes, we addressed the question whether the exogenous gene could itself be regulated. The strain harboring *KlRPL22* in the *RPL22A* locus was transformed with pVTU260 plasmids overproducing either Rpl22A or Rpl22B. Each of the constructs led to the downregulation of *KlRPL22* mRNA to about 40%. In a strain lacking an intron (i.e., where the *RPL22A* intron and exon 2 were replaced by *KlRPL22* exon 2 only), overexpression of the Rpl22A or Rpl22B protein did not reduce *KlRPL22* mRNA abundance.

To prove that the mechanism of *RPL22* regulation is conserved in *K*. *lactis*, we investigated whether *KlRPL22* would respond to Rpl22 overproduction in *K*. *lactis* cells. We observed that plasmid-driven expression of *RPL22A*, *RPL22B*, and *KlRPL22* caused a reduction in *KlRPL22* mRNA levels (Fig 6D). Mutated versions of *RPL22A* or *RPL22B* had no effect, proving that the inhibition is dependent on the intact RNA-binding capacity of Rpl22. These results show that the *KlRPL22* singleton of *K*. *lactis* is capable of intragenic regulation.

### Transcriptome analysis of *rpl22* mutants

**Fig 7:**
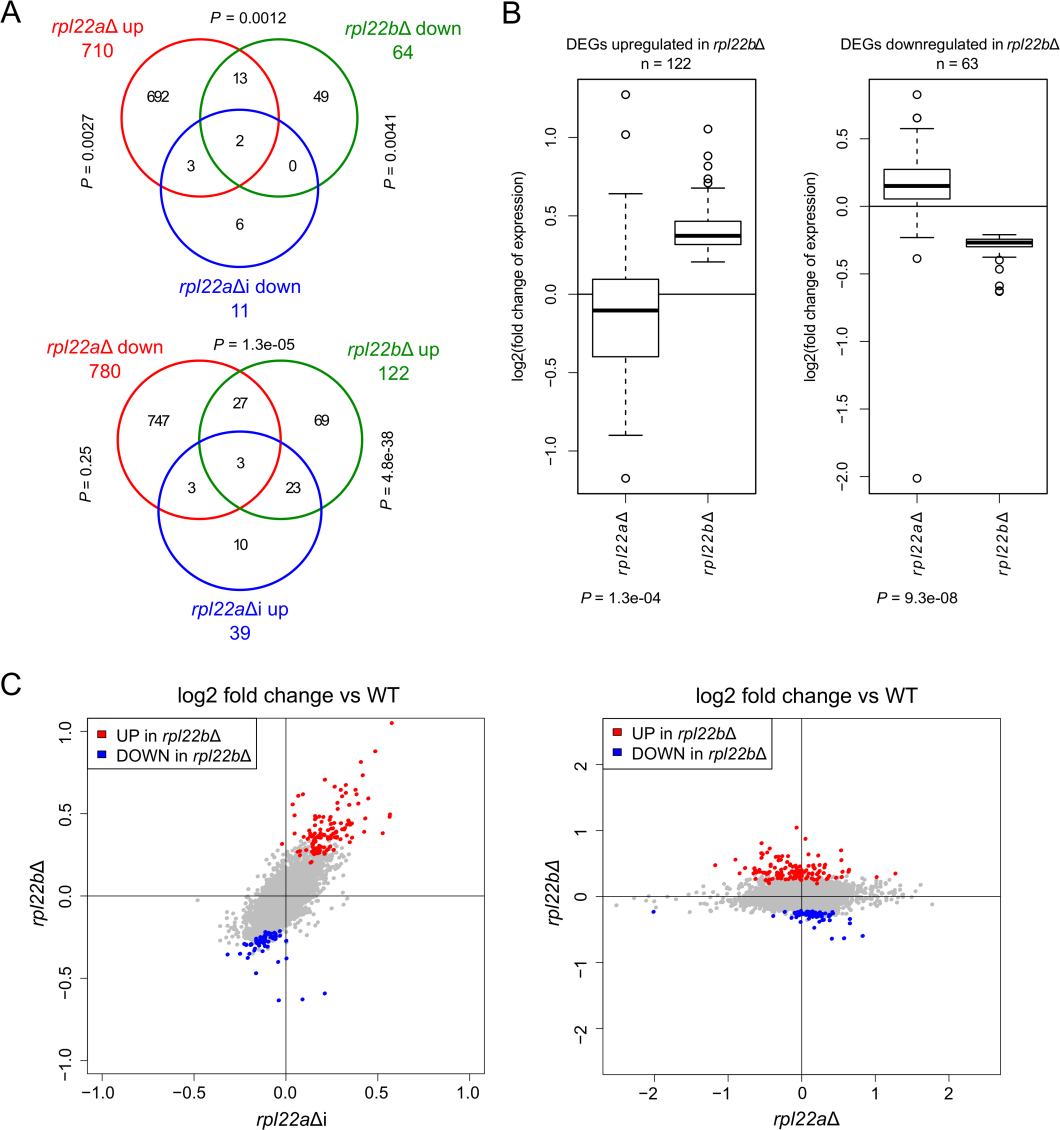
Transcriptome analysis of *RPL22* mutants. (A) Venn diagrams summarising numbers of differentially regulated genes (DEGs) in *RPL22* mutants as compared to WT and showing overlaps between selected sets of *RPL22* mutant DEGs. Overlap significance was determined by Fisher’s exact test. (B) Genes significantly upregulated (left panel) or downregulated (right panel) in *rpl22b*Δ typically show antagonistic regulation in *rpl22a*Δ. Significance was determined by one-sided Wilcoxon signed rank test (*P* value is given below each *rpl22a*Δ panel). Strain genotypes are indicated below the boxplots. The *RPL22B* gene itself was excluded from this analysis (right panel). (C) The DEGs downregulated in *rpl22b*Δ as compared to WT (blue data points) are less affected in *rpl22a*Δ*i* than in *rpl22b*Δ, however their expression changes in the same direction in both mutants. The fold change versus WT of this group of DEGs thus correlates with the negative change of Rpl22B expression (0.1% in *rpl22a*Δ*i* to zero in *rpl22b*Δ) but not with the change of total Rpl22 (see the right panel). The *RPL22A* and *RPL22B* genes themselves were excluded from this analysis.

We measured the impact of *RPL22* gene manipulations at the transcriptome level in order to determine other potential (non-*RPL22*) regulatory intron targets and paralog-specific changes in mRNAs. We performed RNA-Seq analysis of transcriptomes from WT, *rpl22a*Δ and *rpl22b*Δ knock-out, and *rpl22a*Δ*i* and *rpl22b*Δ*i* intron deletion strains. The results are summarized in Fig 7. We observed a much more pronounced change in the transcriptome of *rpl22a*Δ compared to *rpl22b*Δ (1490 vs 186 significant differentially expressed genes (DEGs); Fig 7A and S8 Table). This is in accordance with the consensus that Rpl22A is the major paralog, reflecting the severity of growth defects in the individual *rpl22* knock-out strains (the doubling times in YPD of *rpl22a*Δ, *rpl22b*Δ, and WT are 150.2 min, 90.2 min, and 90.7 min, respectively) [18]. When introns were deleted from the *RPL22* genes, 50 DEGs were identified in *rpl22a*Δ*i*, but only 2 DEGs in *rpl22b*Δ*i* (upregulated *RPL22B* and downregulated *RPL22A*; Fig 7A). Interestingly, manipulating the expression of the two *RPL22* paralogs had antagonistic effects on the transcriptome. These were partial, but significant, overlaps between DEGs upregulated in *rpl22a*Δ and DEGs downregulated in *rpl22b*Δ, and vice versa (Fig 7A). Furthermore, when we reviewed all 186 DEGs up- or downregulated in *rpl22b*Δ and examined their expression in *rpl22a*Δ, these genes typically showed antagonistic regulation between the two *rpl22* deletion strains (Fig 7B and 7C). The RNA-Seq data confirmed the dramatic increase of *RPL22B* mRNA in *rpl22a*Δ cells, but did not reveal additional genes as candidates for direct Rpl22A-dependent and intron-mediated inhibition.

**Fig 8:**
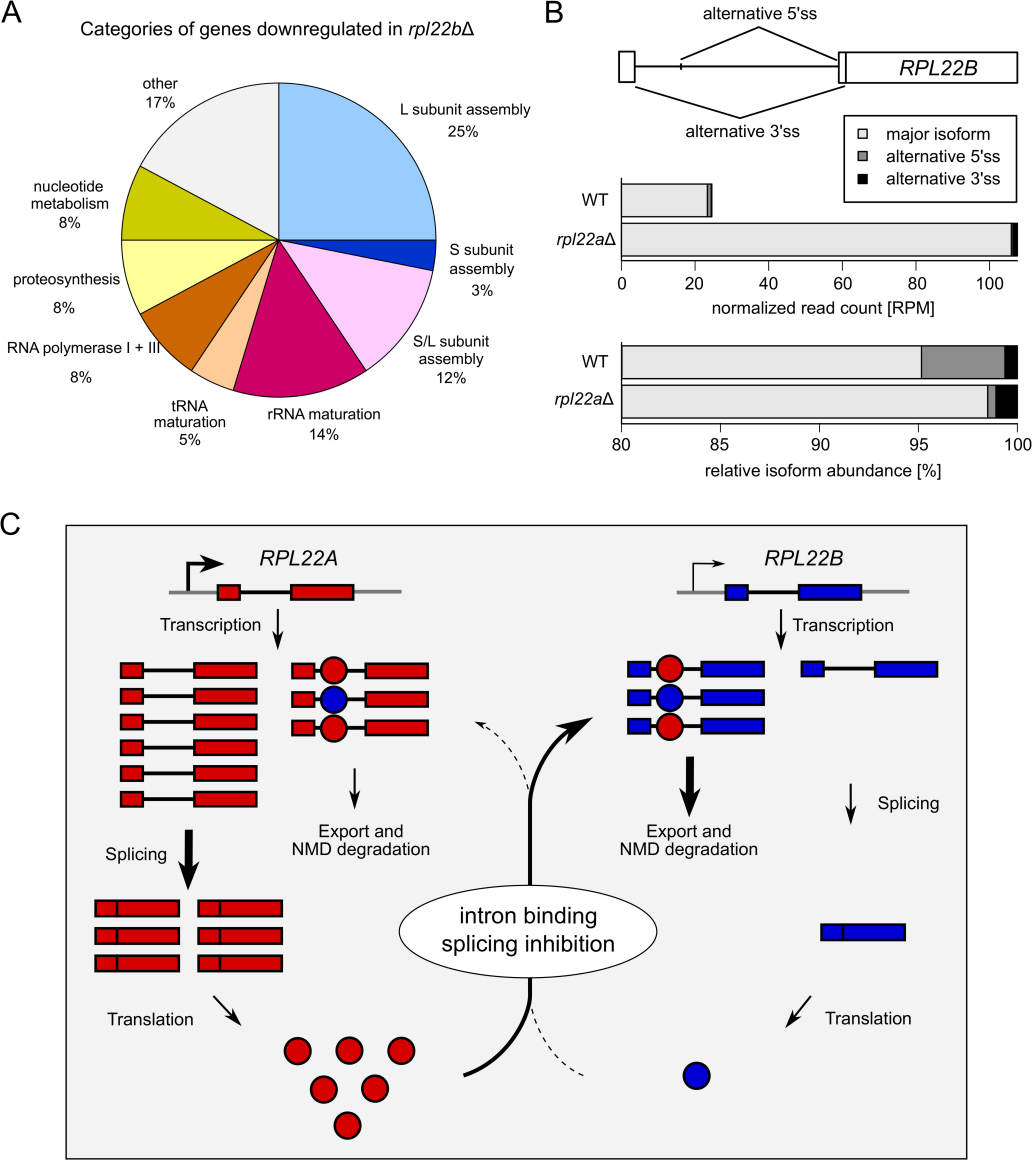
Intergenicaly regulated *RPL22* paralogs may have differentiated roles. (A) Deletion of *RPL22B* has little effect on total *RPL22* levels but affects a subset of genes involved in ribosome biogenesis and proteosynthesis. (B) The scheme depicts alternative splice sites used in *RPL22B* pre-mRNA splicing. The upper plot shows read counts for each spliced *RPL22B* product, normalized to sequencing library sizes (RPM, reads per million). The lower plot shows the percentage of each spliced isoform. Data from pooled samples, adjusted for the number of biological replicates, are shown (n WT = 4, n *rpl22a* = 3). (C) Model summarizing the regulatory interrelationship between *RPL22* paralogs. We assume that both Rpl22 proteins can inhibit splicing of either *RPL22A* or *RPL22B* pre-mRNA. Splicing of *RPL22B* pre-mRNA is much more sensitive to the inhibitory effect of Rpl22. This model represents the situation in WT cells, where Rpl22A is the major Rpl22 protein. Thus, its level contributes considerably to the splicing regulation of the minor *RPL22B* paralog but also partially to controlling its own expression. The Rpl22B level is low but can still provide minor regulation of its own as well as Rpl22A production.

We estimated the splicing efficiencies of endogenous *RPL22* genes from our RNA-Seq data using a method described previously [75]. The splicing efficiency changes in the manipulated strains were in agreement with qPCR measurements and splicing reporter results. Low levels of Rpl22 protein in *rpl22a*Δ facilitated unrestrained splicing of *RPL22B*, as also evidenced by lower pre-mRNA levels (see S1 Fig). Interestingly, this was accompanied by changes in the proportions of two alternatively spliced minor isoforms, which we identified in the RNA-Seq data (Fig 8B). The 5’ss-alternative isoform was produced with higher frequency in WT (where the splicing of *RPL22B* was partly inhibited) than in *rpl22a*Δ (where the inhibition was lifted). The 3’ss-alternative isoform, which shares 5’ss with the major product, behaved similarly to the major product. While the existence of these alternative isoforms (both of which contain STOP codons) has been reported previously [82], their differential behavior with respect to Rpl22 inhibition is here documented for the first time.

To better understand the specific processes affected by the Rpl22 paralogs, we performed GO enrichment analysis of the respective sets of up- and downregulated DEGs (S9 Table). We found that ribosome biogenesis genes were enriched both among DEGs upregulated in *rpl22a*Δ and among DEGs downregulated in *rpl22b*Δ. Also, DEGs upregulated in *rpl22b*Δ or *rpl22a*Δ*i* were enriched for genes involved in sugar metabolism (mostly trehalose synthesis) and in phosphate metabolism. Furthermore, *rpl22a*Δ DEGs were specifically enriched for genes involved in mitochondrion organization (downregulated) and amino acid metabolism (upregulated). On the other hand, the deletion of *RPL22B* led to the upregulation of stress-response genes and the downregulation of factors important for ribosome biogenesis (different from those upregulated in *rpl22a*Δ; Fig 8A). The data suggest that the minor *RPL22B* acquired a paralog-specific role, as its deletion produces a distinct transcription phenotype at ~WT total *RPL22* levels.

## Discussion

Deciphering the physiological significance of the RPG regulon of *S*. *cerevisiae* may contribute toward our understanding of how the underlying framework of the genome is used in evolutionary adaptations [83]. The selection of strains that best respond to the contradictory challenges of stressors and nutrients is perhaps the reason for the high retention rate of paralogs among yeast RPGs [3,84]. The panel of nuclear-encoded mitoribosomal protein genes does not contain ohnologs [85,86]. The number of proteins translated in mitochondria is limited but, perhaps more importantly, it was the adaptation to anaerobic environments, such as insect guts, which drove this ohnolog-based strategy [81]. RPG transcripts differ markedly in their untranslated regions and the nucleotide diversity in the 5’-UTRs of RPGs is higher in comparison to housekeeping genes [20]. Duplicated genes contribute more resistance to deleterious mutations not only because the two alleles can substitute for each other, but also because the regulatory network is more able to respond by rewiring [87,88]. More variability in regulatory regions means a more diversified protein interactome [89] and better adaptivity of the network as a whole [90]. Such adaptations may require paralog pairs that differ only in regulatory parts of genes or by one or two amino acids, as is the case for 42 of the 59 RPG duplexes. One exciting possibility is that paralogous genes generate proteins that are paralog-specifically modified. Their posttranslational modifications may be executed differentially based on differences in untranslated regions and/or translation efficiencies between paralogous mRNAs [91]. Remarkably, human RPGs cannot substitute for their yeast counterparts [92] despite the high degree of amino acid sequence conservation, which suggests the existence of yeast-specific network-based adaptations. While the high retention rate of introns attests to their importance for organisms, the underlying reason for this retention warrants further research.

### Intergenic regulation of *RPL22A/B* is splicing-mediated and asymmetric

We monitored mRNA and pre-mRNA levels in strains with various combinations of intron and whole gene deletions in *RPL22A* and *RPL22B* paralogs (Fig 1). While *RPL22A* mRNA ranged from 160% to 60% of WT, the amplitude for *RPL22B* was much larger (500% to 5% of *RPL22B* WT), which suggests that *RPL22B* concentration provides the “readout” for regulatory purposes. Our results are in agreement with the research carried out by Gabunilas and Chanfreau [59] and were obtained independently of their results using partially different methods.

Proteomic analysis of the manipulated strains shows here for the first time that Rpl22A/Rpl22B protein levels match the ratios of corresponding mRNAs (Fig 1C). Using splicing reporters (Fig 1) as well as knock-ins of chimeras in the *RPL22* loci (Fig 4), we demonstrate that splicing of the *RPL22B* intron is stringently regulated by Rpl22 levels, whereas the *RPL22A* reporter is much less sensitive (Fig 1). The reporters contained endogenous intron sequences as well as parts of 5’-UTR, exon 1, and part of exon 2. These results complement the data of Gabunilas and Chanfreau [59], where constructs used to measure splicing lacked alternative 5’ss and contained only intronic sequences. Our results are also in agreement with the conclusion that *RPL22A/B* regulation occurs at the pre-mRNA splicing step. This does not exclude the possibility that, under stress or under conditions affecting growth, more complex regulation (involving translation efficiency [55], or protein stability) takes place.

In exponentially growing WT cells, splicing of both paralogs is apparently subject to negative regulation by Rpl22 (Figs 1 and 3). Therefore, free Rpl22 concentration must be well above dissociation constant for *RPL22B* intron binding. Under conditions of balanced RP production and ribosome biogenesis, Rpl22 concentration in the nucleoplasm is determined by the pool of free Rpl22 [93] and by the affinities of Rpl22 for its binding sites on large subunits or their maturation stages. A sudden excess of free Rpl22 would mildly inhibit *RPL22A* splicing and further lower the expression of Rpl22B (i.e., the B/A ratio would become very low). According to our findings, this is the situation which affects the transcription of genes involved in ribosome biogenesis (Fig 8). It is possible that the fluctuations in free Rpl22B concentrations help to fine-tune ribosome assembly during disturbances caused by nutrition or environmental changes.

Intron mediated-regulation is insensitive to Rpl22A/B amino acid differences and both Rpl22 proteins may bind to a structured region between 5’ss and BP in the *RPL22B* intron when assayed in the yeast three-hybrid system (I2; Fig 5). In contrast, the 3’ss-proximal hairpin structure in the *RPL22A* intron (S2 Fig) had no role in splicing regulation (S1 Fig). In all instances documented thus far, the extraribosomal functions of Rpl22 (see Introduction) are mediated through RNA binding, most likely involving secondary transcript structures and the rRNA-binding domain of the protein. Human Rpl22 has been shown to bind to a broadly defined short hairpin loop (5’-NNNNG (N)_7_ CUNNN-3’) [94], which it can target within its own mRNA, the intron of Smad2, or the human Epstein Barr virus, EBER-1 RNA [48–50]. The secondary structures mediating Rpl22 inhibition in the *RPL22B* and *RPL22A* introns warrant further study. There is no obvious similarity to the rRNA structure or the SELEX motif identified for the human protein [94,95]. However, given the three-hybrid results (Fig 5) and the fact that RNA-binding mutants lack inhibitory potential (see Figs 3 and 4), there is a strong indication that intron RNA forms a structure that can be recognized by Rpl22. The situation may be more complex, as the structure can be complemented by other proteins or RNA, such as parts of the intron. Alternatively, it may comply with Rpl22-binding requirements, despite the lack of any apparent sequence similarity. Intriguingly, the intron-Rpl22 interaction specifically inhibited usage of the major, but not the minor, 5’ss (Fig 8B).

We observed ~4-8-fold increases in *RPL22A/RPL22B* pre-mRNA levels in the mutants defective for the cytoplasmic NMD pathway and 5’-3’ degradation (Fig 2, S3 Fig). This indicates that *RPL22* pre-mRNAs are unable to compete for the splicing machinery in the presence of Rpl22. The data also imply that the inhibitory complexes containing the pre-mRNAs are stable until the time the pre-mRNAs are subjected to NMD in the cytoplasm. Our observations extend the data of Gabunilas and Chanfreau [59]. Unlike these authors, we observed no tendency for an increase of *RPL22* mRNA in the *XRN1* mutant, which may perhaps reflect the cultivation conditions-dependent availability of free Rpl22.

We summarize that splicing inhibition of *RPL22A/RPL22B* is operational at both introns, albeit to a different degree, and that it is dependent on Rpl22 RNA-binding propensity but independent of the differences in 19 amino acids between the Rpl22A and Rpl22B proteins. These results both support the conclusions drawn by Gabunilas and Chanfreau [59] and further extend their findings. We were able to demonstrate the effects of ectopically produced Rpl22A/B proteins in the *rpl22a*Δ*b*Δ background. We also showed that RNA-binding mutant versions of Rpl22 are devoid of regulatory activity. Using the yeast three-hybrid system, we located Rpl22 binding to a region which overlaps with the fragment described by Gabunilas and Chanfreau [59].

### Intron-dependent regulation of *S*. *cerevisiae RPL22* most likely evolved from autoregulation

*Kluyveromyces lactis* is part of the “*Saccharomyces* complex” (which did not undergo WGD) and has a single-copy *RPL22* homolog [8]. The alignment of *RPL22A*, *RPL22B*, and *KlRPL22* shows that all three introns share a common motif (S2 File, underlined), which is also present in the *RPL22* introns of other *Saccharomycotina* species (not shown; [59]). This motif (i) lies partly within the I2 construct that interacts with Rpl22 in the three-hybrid system (Fig 5) and (ii) borders the element important for splicing inhibition identified by Gabunilas and Chanfreau [59]. We assayed the regulatory properties of the *K*. *lactis* intron and protein in both *S*. *cerevisiae* and *K*. *lactis*. When the *K*. *lactis* intron was introduced together with exon 2 into the *S*. *cerevisiae RPL22A* locus, its splicing was repressed at high Rpl22 protein levels similar to the endogenous *RPL22A* intron. We also showed that while the KlRpl22 protein was capable of regulating *RPL22A* and *RPL22B* in *S*. *cerevisiae* (Fig 6A and 6B), Rpl22A/B as well as KlRpl22 inhibited splicing of *KlRPL22* in *K*. *lactis* (Fig 6D). These results prove that *K*. *lactis RPL22* may be regulated by an intragenic loop, whereby the KlRpl22 protein level modulates splicing of its own transcript. If we assume that a similar situation existed in the ancestor of *S*. *cerevisiae* before WGD, then, hypothetically, such autoregulation would have become intergenic and asymmetric after WGD (see Fig 8C). Therefore, the basis of the *RPL22* (auto)-regulatory mechanism is older than the difference between the *S*. *cerevisiae* Rpl22 proteins. We contend that Rpl22 acquired its intron-dependent function in the lineage ancestral to the “*Saccharomyces* complex”.

Rpl22A and B differ from KlRpl22 by 14 and 23 amino acids, respectively, and from each other by 19 amino acids, with most of the differences in the C-terminal part. The differences between A and B seem not to affect the capacity of the protein to inhibit splicing of either intron (see Figs 1, 3 and 4). KlRpl22, which is more similar to Rpl22A, is also capable of recognizing *S*. *cerevisiae* pre-mRNAs. The degree of inhibition of *KlRPL22* splicing is more similar to the responsiveness of *RPL22A* (to ~40%). We speculate that after WGD the expression of the genes eventually became asymmetric, turning *RPL22B* into a minor paralog. We also hypothesize that splicing-mediated regulation might have contributed to the establishment of the asymmetry. Apparently, only after the duplication was a stringent inhibition (of one of the introns) enabled. It is notable that both *RPL22A* and *RPL22B* contain the non-canonical 5’ss sequences, GTATGA and GTACGT, respectively, which are conserved across the *S*. *cerevisiae sensu stricto* group. The singleton *KlRPL22* contains a canonical site. A similar situation exists in the meiotic *MER1* regulon, where Mer1-regulated genes retain non-consensus splice sites across the species of the group [96]. Another feature of asymmetry between *RPL22* paralogs is the presence of two Rap1 binding sites in their 5’-regulatory region. *RPL22B* apparently can only use one, as its 3’-proximal site has a C/A mutation of one of the two key cytosines within the binding consensus (CACCCATACAT [97]).

The regulation of *KlRPL22* poses a question whether the *K*. *lactis* singletons corresponding to the other regulated RPG pairs are always regulated in autogenic fashion. We intend to test this possibility in future experiments. While *RPL22A*/*B* pair and its kin might represent autogenic regulation turned intergenic, other RPs might have acquired their extraribosomal roles (i.e., RNA structures as binding partners) only after WGD. *RPL2A* (see Fig 1A), which seems to be regulated in a paralog-specific autogenic mode, is a candidate for such a scenario.

### Genome-wide effects of *RPL22* manipulations

We were intrigued by the possibility of other nonribosomal-RNA regulatory targets for RPs [49]. Rpl22 may regulate the splicing of other introns, such as those of other RPGs, in addition to acting on its own gene and paralog. We therefore decided to compare transcriptome changes in situations with differing paralog ratios. While we did not find changes that would indicate additional intron-containing regulatory targets of Rpl22, our data illustrate the requirement of Rpl22 for ribosome biogenesis and also include DEGs involved in metabolism. They are also in partial agreement with previously reported Affymetrix expression microarray profiles of *rpl22a*Δ that include amino acid and carboxylic acid metabolism among the upregulated DEG categories [22]. Previously, Rpl22 has been implicated in cell-fate decisions in response to environmental signals [55]. Cells with reduced Rpl22 levels have been shown to exhibit a reduced 60S:40S particle ratio and accumulated “halfmers” on polysomes, which indicates that mRNA-bound 40S particles lack their 60S counterparts [58]. *RPL22A* is also among the RPs that affect the replicative lifespan and confer resistance to tunicamycin-induced ER stress [18].

Deletion of *RPL22B*, which leaves cells with A-only Rpl22 at the ~WT mRNA level, up-/downregulated 122/63 DEGs. We found this group of transcripts very interesting because they represent a response to the removal of the Rpl22B paralog without deviating from the ~WT Rpl22 level (Fig 1). The downregulated DEGs exhibited factors involved in the biogenesis of both ribosomal subunits, rRNA metabolism, subunits of RNA Pol I and III, nucleotide salvage and synthesis pathway genes, tRNA modification factors, and factors involved in proteosynthesis. The upregulated DEGs contained genes that are involved in stress response and metabolism, including nucleotide and carbohydrate metabolism. *RPL22A* intron deletion, which downregulates *RPL22B* and upregulates total *RPL22* concentration, yielded a substantial overlap with the *rpl22b*Δ phenotype (Fig 7A). Our data thus suggest that Rpl22B assumes a paralog-specific role that affects ribosome biogenesis.

RPG pairs coding for different protein isoforms can be used to modify ribosome composition and function (see Introduction). *RPL22* appears as a pair of paralogs in unrelated taxa analogically to, for example, *RPS9* [13]. In such cases, subfunctionalization involves proteins as well as UTRs and introns. The differences can affect Rpl22 functions during ribosome biogenesis and/or during translation within the large ribosomal subunit [22,25,58] or they may be related to the extraribosomal tasks of Rpl22 proteins.

Gabunilas and Chanfreau report that MMS-induced DNA stress or disruption of rRNA transcription causes splicing-mediated downregulation of *RPL22B* mRNA (and Rpl22B production) [59]. Such effects downregulate ribosome biogenesis, which should increase free Rpl22 levels and thus *RPL22B* inhibition (see the model in Fig 8C). The early response to oxidative stress may also involve changes in translation efficiency of specific transcripts that are enriched for TTG (Leu) codons [55], including *RPL22A*. The translation effect is brought about by Tmr4 methyltransferase, which produces tRNA^LEU(CAA)^ with methylated C at the wobble position. This modification specifically potentiates anticodon-codon binding, thus enhancing the translation of some, albeit not all, TTG-enriched mRNAs [55,98]. While *RPL22A* is TTG enriched, *RPL22B* is not. In fact, *RPL22B* is the most distant codon-usage outlier of the cytoplasmic RPGs [99]. Hypothetically, the Rpl22A/B ratio may thus predetermine the amplitude and character of the reaction to oxidative stress. The immediate-early translation response requires *RPL22A* (and further enhances the excess of A over B). This should synergize with the inhibition of biogenesis (see Fig 8A). Restarted ribosome biogenesis should lead to decreased levels of RPs (including Rpl22) in the nucleoplasm, eventually unblocking *RPL22B* splicing. Increased Rpl22B should then further enhance ribosome biogenesis.

The extraribosomal roles of RPs [18,100] are far from widespread and not all RPs are equally suited for their tasks. Why is it that Rpl22 (or Rpl30, Rps9, among others [13,41]) binds its own transcript and regulates its splicing whereas other (RNA binding) RPs seem not to do so? We argue that due to the heavy constraint on the variability of the protein with respect to its ribosomal function, it must have been the variability on the RNA level which gave rise to Rpl22 binding structures in the transcriptome. This variability should be similar for all RPGs, however. Perhaps the discerning factor was the position of the protein in a key node that regulates ribosome biogenesis [4,101,102]. Such an intron-RP interaction would have been more likely to be fixed in cell population once its impact on splicing improved fitness [19,20]. Alternatively, it may have been the availability of the protein in the nucleoplasm, because of the association of the protein with the ribosome on its periphery or at a lower affinity, which made it available to sample other interactions (or “evolutionary opportunities” [103]). Evidence for the extraribosomal roles of Rpl22s in divergent taxa throughout evolution perhaps illustrates the exploitation of such opportunities [49,50,104]. It would be of interest to uncover more examples and to learn what predisposes their (re-)occurrence in organisms. Moreover, the regulated splicing of *RPL22A/RPL22B* (see Fig 8C) may serve as a model for establishing a regulatory relationship between two paralogous genes in evolution.

## Supporting information

S1 Fig *RPL22* paralogs show intron-dependent intergenic relationship. Plots show mean fold changes of mRNA and pre-mRNA levels of *RPL22A* (upper panel) and *RPL22B* (lower panel) relative to WT as determined by RT-qPCR. Data were normalized to the *SPT15* expression and to the RNA level in WT cells. n indicates the number of biological replicates and error bars represent s.d.; ND—not determined. This figure complements Fig 1. *P* values were obtained for comparisons between WT strain and a mutant strain using the t-test with Holm correction for multiple testing (see Methods). Only *P<*0.05 (considered statistically significant) are indicated.
(TIF)

S2 Fig Secondary structures of *RPL22A* and *RPL22B* introns. (A) Structure of *RPL22A* intron. Bold line indicates the hairpin structure that forms between branch-point (BP; marked by *) and 3’ splice site (3’ss; marked by arrow). Detailed view shows the structural prediction for the region between BP and 3’ss. Dashed line marks the position where the hairpin was ablated to generate the *rpl22a*ΔH (Δ Hairpin) mutant. (B) Structure of *RPL22A* intron and the detail of BP to 3’ss region after hairpin removal (former hairpin position marked by dashed line). (C) Structure of *RPL22B* intron. All structures were predicted by RNAFold [1]. Color key represents the scale of positional entropy. Red color indicates high stability/probability of the corresponding structure.
(TIF)

S3 Fig *RPL22* expression in double mutant strains carrying *air1*Δ, *air2*Δ, *upf1*Δ, or *dbr1*Δ allele and *RPL22A*/*RPL22B* intron deletion. *RPL22* transcript levels in strains with deletion of *AIR1* (A), *AIR2* (B), *UPF1* (C) and *DBR1* (D) were determined by RT-qPCR. Plots show mean fold change of RNA expression normalized to *SPT15* transcript and WT. Error bars represent s.d. from biological replicates indicated by n.; ND—not determined. The statistical significance of the difference between WT strain and a mutant strain is indicated as (1) for *P≤*0.05, (2) for *P≤*0.01, and (3) for *P≤*0.001 based on the t-test with Holm correction for multiple testing (see Methods).
(TIF)

S4 Fig Gene dependency of pre-mRNA accumulation in response to splicing and RNA degradation pathway mutations. (A) Truncation of the spliceosomal protein Prp45 [2] or deletions of components of the cytoplasmic 5’-3’ RNA degradation pathways, and combinations of these mutations affect mRNA levels of *RPL22A*, *RPL22B*, *ECM33* and *COF1* genes only to a limited extent (with the exception of *xrn1*Δ, which lowers the mRNA level by up to 50% in some cases). (B) The extent of *RPL22A* and *RPL22B* pre-mRNA accumulation in strains mentioned above differs from that of *ECM33* and *COF1* genes. mRNA and pre-mRNA levels were measured by RT-qPCR with primers spanning exon-exon junctions or exon-intron junctions, respectively. Data were normalized to *TOM22* gene and expressed as mean fold change relative to WT level (indicated by a horizontal line). Error bars represent s.d. of two biological replicates for all *RPL22*-related analyses and for *ECM33*- and *COF1*-related analyses in *xrn1*Δ and *xrn1*Δ *prp45*(1–169) strains. In the remaining *ECM33*- and *COF1*-related measurements, error bars represent s.d. of 3 biological replicates. The statistical significance of the difference between WT strain and either *prp45*(1–169) strain or pooled data (indicated by horizontal bolts) of NMD mutants or NMD *prp45*(1–169) double mutants was analyzed by t-test with Holm correction for multiple testing (see Methods). Only *P<*0.05 (considered statistically significant) are indicated.
(TIF)

S5 Fig Western blots of Rpl22A/Rpl22B and their RNA binding mutants in the strains used in this study. Production of 6xHIS-tagged Rpl22 variants was confirmed by Western blot in strains used for the analyses of Rpl22 effect on *RPL22* RNA (A) expression, and (B) splicing efficiency by primer extension. Anti-PSTAIR antibody was used as a loading control.
(TIF)

S6 Fig Western blots of Rpl22A/Rpl22B and their RNA binding mutant hybrid proteins produced in the three-hybrid YBZ1 strain. The production of Rpl22A, Rpl22B and their RNA binding mutants, fused with HA-Gal4 activation domain, was verified using Western blot analysis with anti-HA antibody. Hybrid proteins of 31 kDa (marked with arrow) were detected in extracts from all strains tested in three-hybrid system. M—protein size marker.
(TIF)

S1 Table List of strains used in this study. (XLSX)

S2 Table List of oligonucleotides used in this study. (XLSX)

S3 Table List of plasmids used in this study. (XLSX)

S4 Table Combinations of oligonucleotides used for intron and/or gene replacements. (XLSX)

S5 Table Combinations of primers used for copy-specific detection of *RPL22* in intron and/or gene manipulated strains. (XLSX)

S6 Table Numeric rendering of heatmaps in Fig 3A. (PDF)

S7 Table Numeric rendering of heatmaps in Fig 4. (PDF)

S8 Table DEGs. (XLS)

S9 Table Go enrichment. (XLS)

S1 File Supplementary methods and references. (DOCX)

S2 File Alignment of *RPL22* introns and proteins from *S*. *cerevisiae* and *K*. *lactis*. (DOCX)
