# Supplementary figures and images for: Introns provide a platform for intergenic regulatory feedback of RPL22 paralogs in yeast
Source: PLoS One. 2018 Jan 5;13(1):e0190685. doi: 10.1371/journal.pone.0190685 (PMC5755908; doi:10.1371/journal.pone.0190685)

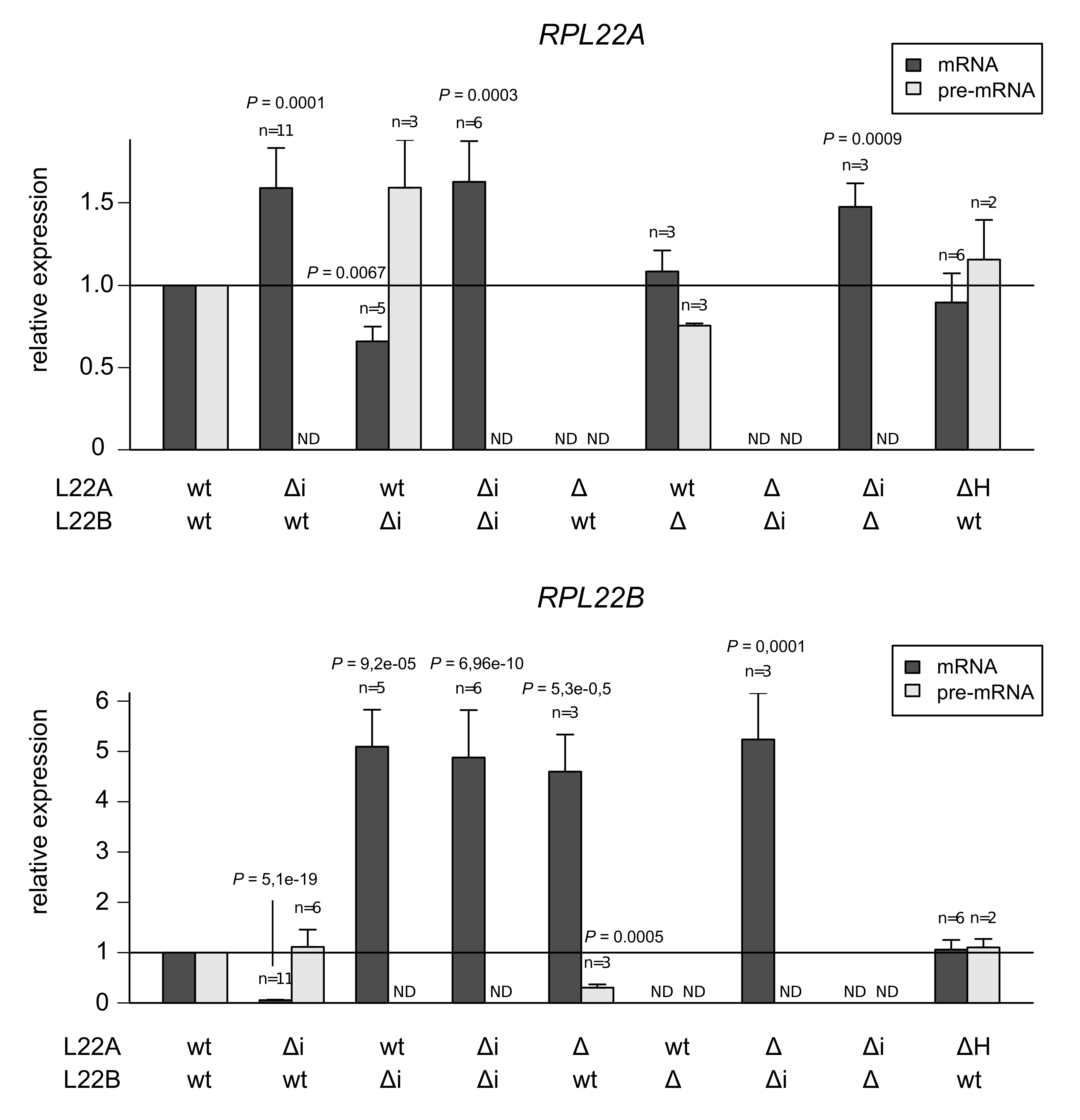

Supplement: S1 Fig — Plots show mean fold changes of mRNA and pre-mRNA levels of RPL22A (upper panel) and RPL22B (lower panel) relative to WT as determined by RT-qPCR. Data were normalized to the SPT15 expression and to the RNA level in WT cells. n indicates the number of biological replicates and error bars represent s.d.; ND—not determined. This figure complements Fig 1. P values were obtained for comparisons between WT strain and a mutant strain using the t-test with Holm correction for multiple testing (see Methods). Only P<0.05 (considered statistically significant) are indicated. (TIF) [file pone.0190685.s001.tif]

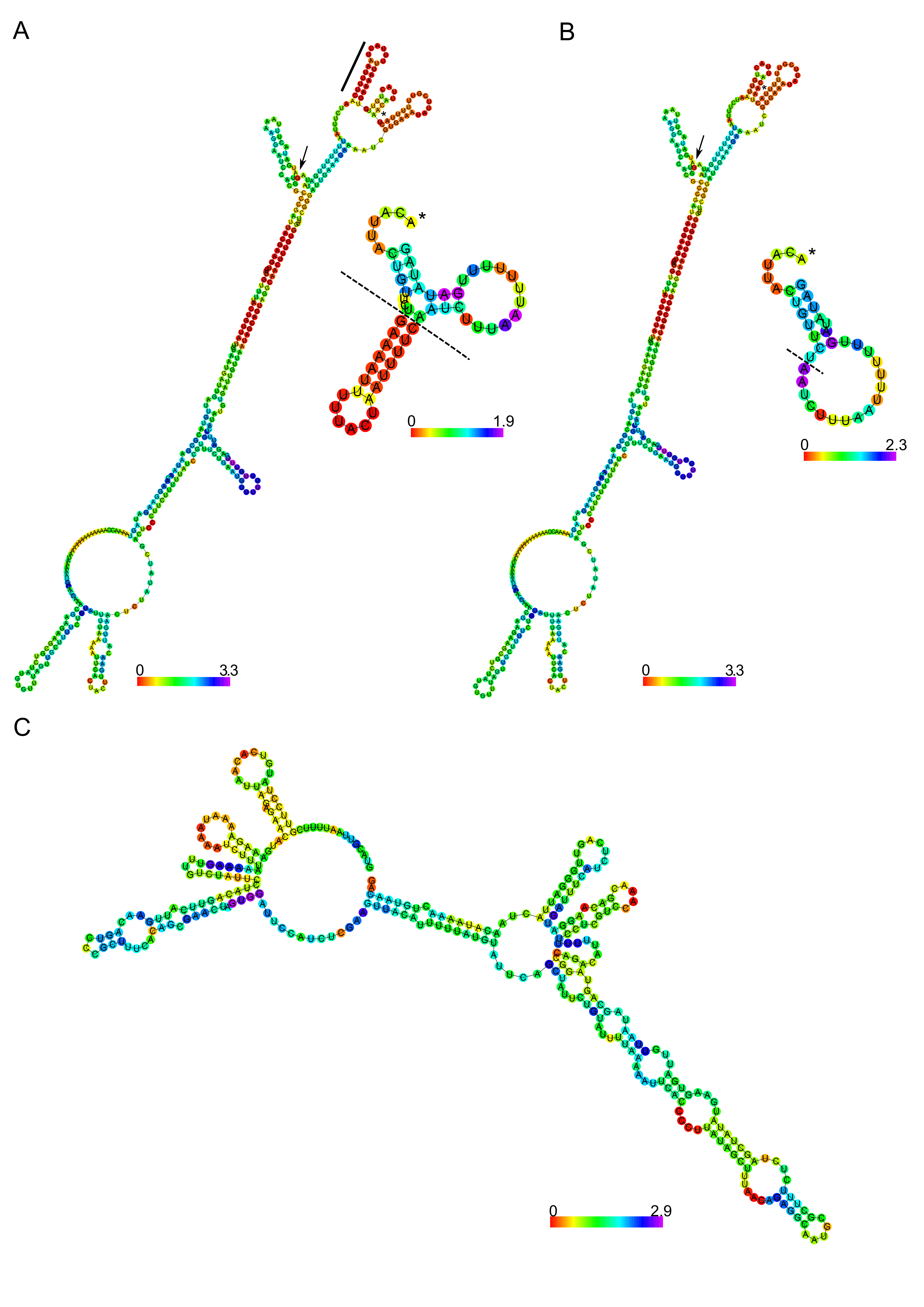

Supplement: S2 Fig — (A) Structure of RPL22A intron. Bold line indicates the hairpin structure that forms between branch-point (BP; marked by *) and 3’ splice site (3’ss; marked by arrow). Detailed view shows the structural prediction for the region between BP and 3’ss. Dashed line marks the position where the hairpin was ablated to generate the rpl22aΔH (Δ Hairpin) mutant. (B) Structure of RPL22A intron and the detail of BP to 3’ss region after hairpin removal (former hairpin position marked by dashed line). (C) Structure of RPL22B intron. All structures were predicted by RNAFold [1]. Color key represents the scale of positional entropy. Red color indicates high stability/probability of the corresponding structure. (TIF) [file pone.0190685.s002.tif]

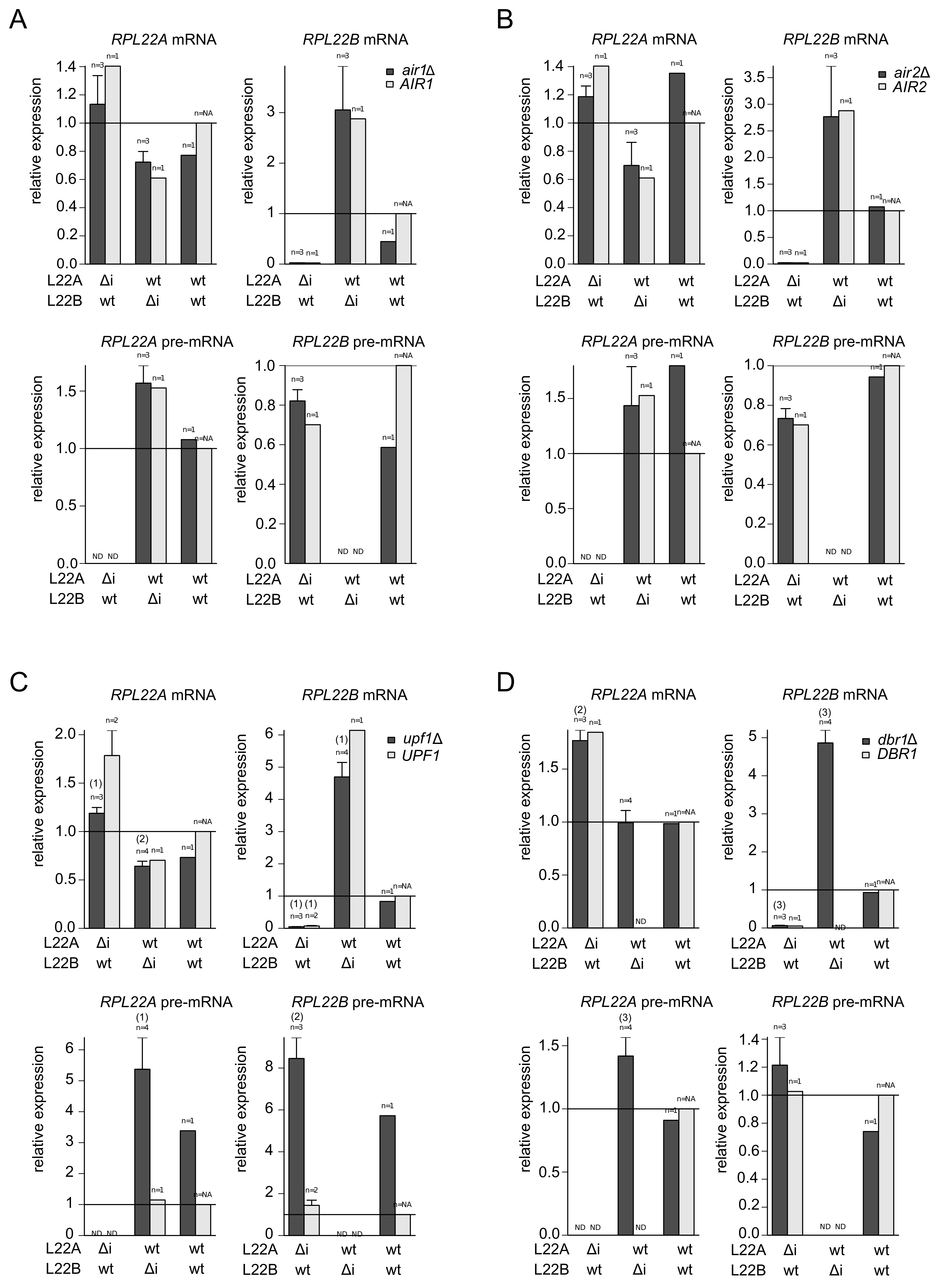

Supplement: S3 Fig — RPL22 transcript levels in strains with deletion of AIR1 (A), AIR2 (B), UPF1 (C) and DBR1 (D) were determined by RT-qPCR. Plots show mean fold change of RNA expression normalized to SPT15 transcript and WT. Error bars represent s.d. from biological replicates indicated by n.; ND—not determined. The statistical significance of the difference between WT strain and a mutant strain is indicated as (1) for P≤0.05, (2) for P≤0.01, and (3) for P≤0.001 based on the t-test with Holm correction for multiple testing (see Methods). (TIF) [file pone.0190685.s003.tif]

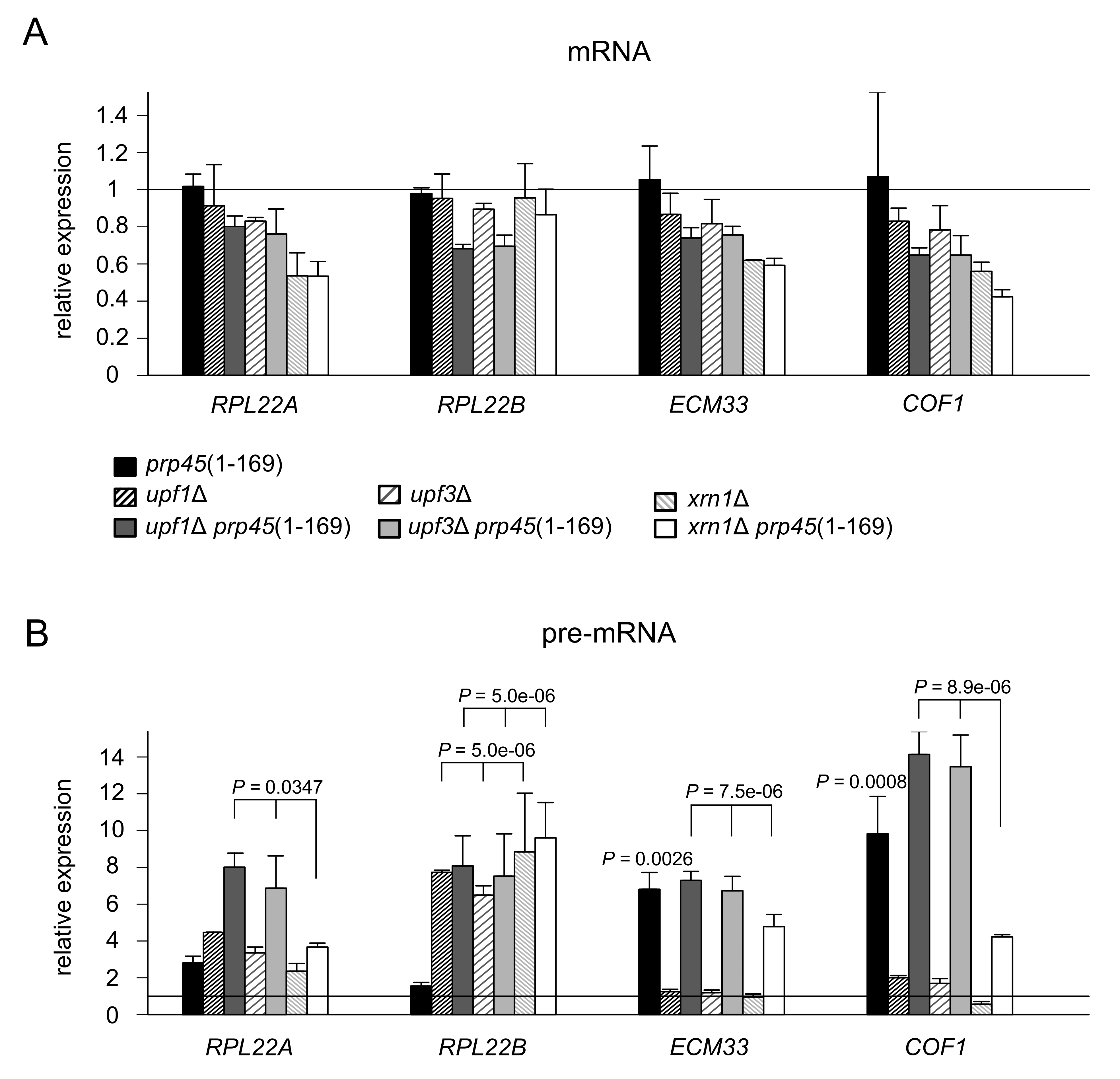

Supplement: S4 Fig — (A) Truncation of the spliceosomal protein Prp45 [2] or deletions of components of the cytoplasmic 5’-3’ RNA degradation pathways, and combinations of these mutations affect mRNA levels of RPL22A, RPL22B, ECM33 and COF1 genes only to a limited extent (with the exception of xrn1Δ, which lowers the mRNA level by up to 50% in some cases). (B) The extent of RPL22A and RPL22B pre-mRNA accumulation in strains mentioned above differs from that of ECM33 and COF1 genes. mRNA and pre-mRNA levels were measured by RT-qPCR with primers spanning exon-exon junctions or exon-intron junctions, respectively. Data were normalized to TOM22 gene and expressed as mean fold change relative to WT level (indicated by a horizontal line). Error bars represent s.d. of two biological replicates for all RPL22-related analyses and for ECM33- and COF1-related analyses in xrn1Δ and xrn1Δ prp45(1–169) strains. In the remaining ECM33- and COF1-related measurements, error bars represent s.d. of 3 biological replicates. The statistical significance of the difference between WT strain and either prp45(1–169) strain or pooled data (indicated by horizontal bolts) of NMD mutants or NMD prp45(1–169) double mutants was analyzed by t-test with Holm correction for multiple testing (see Methods). Only P<0.05 (considered statistically significant) are indicated. (TIF) [file pone.0190685.s004.tif]

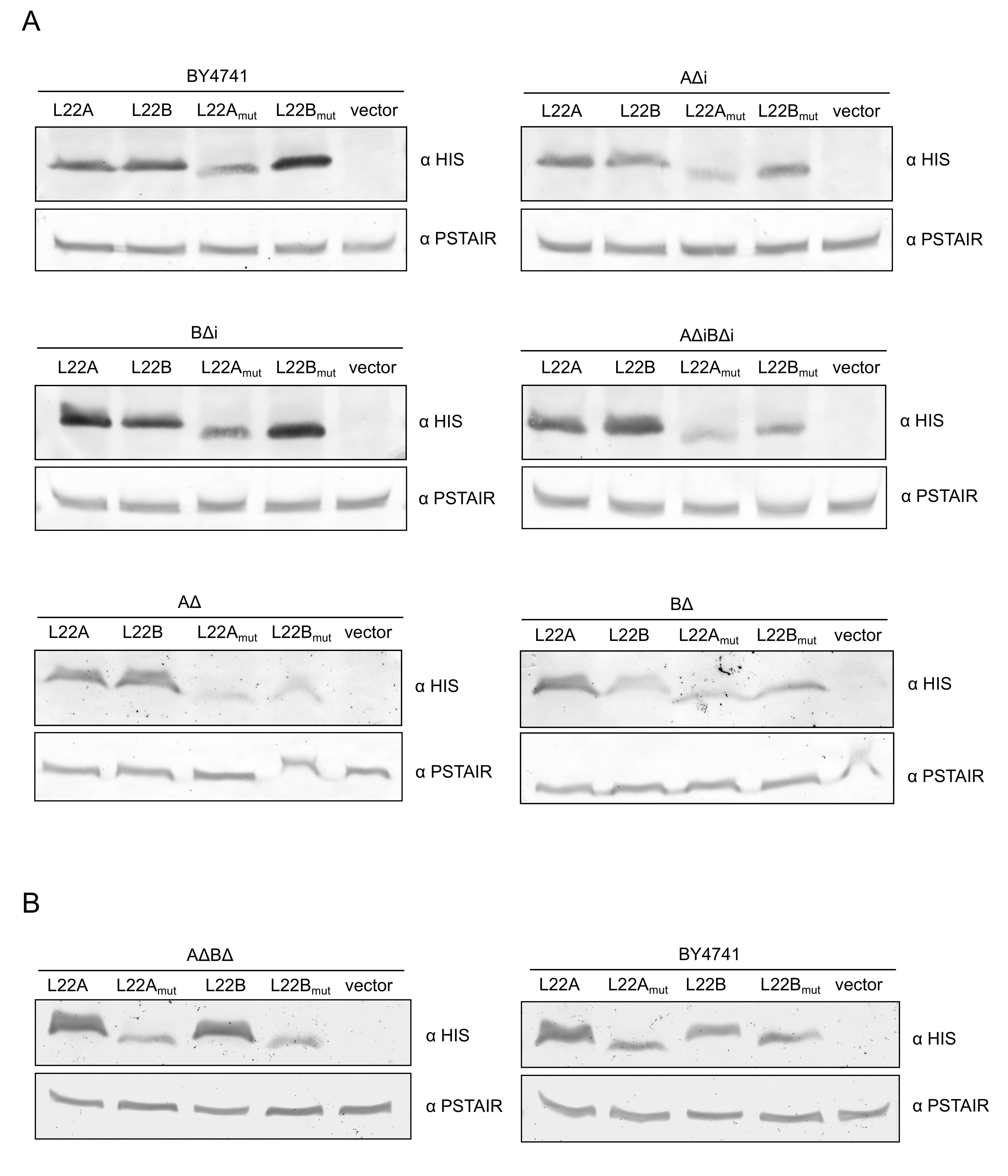

Supplement: S5 Fig — Production of 6xHIS-tagged Rpl22 variants was confirmed by Western blot in strains used for the analyses of Rpl22 effect on RPL22 RNA (A) expression, and (B) splicing efficiency by primer extension. Anti-PSTAIR antibody was used as a loading control. (TIF) [file pone.0190685.s005.tif]

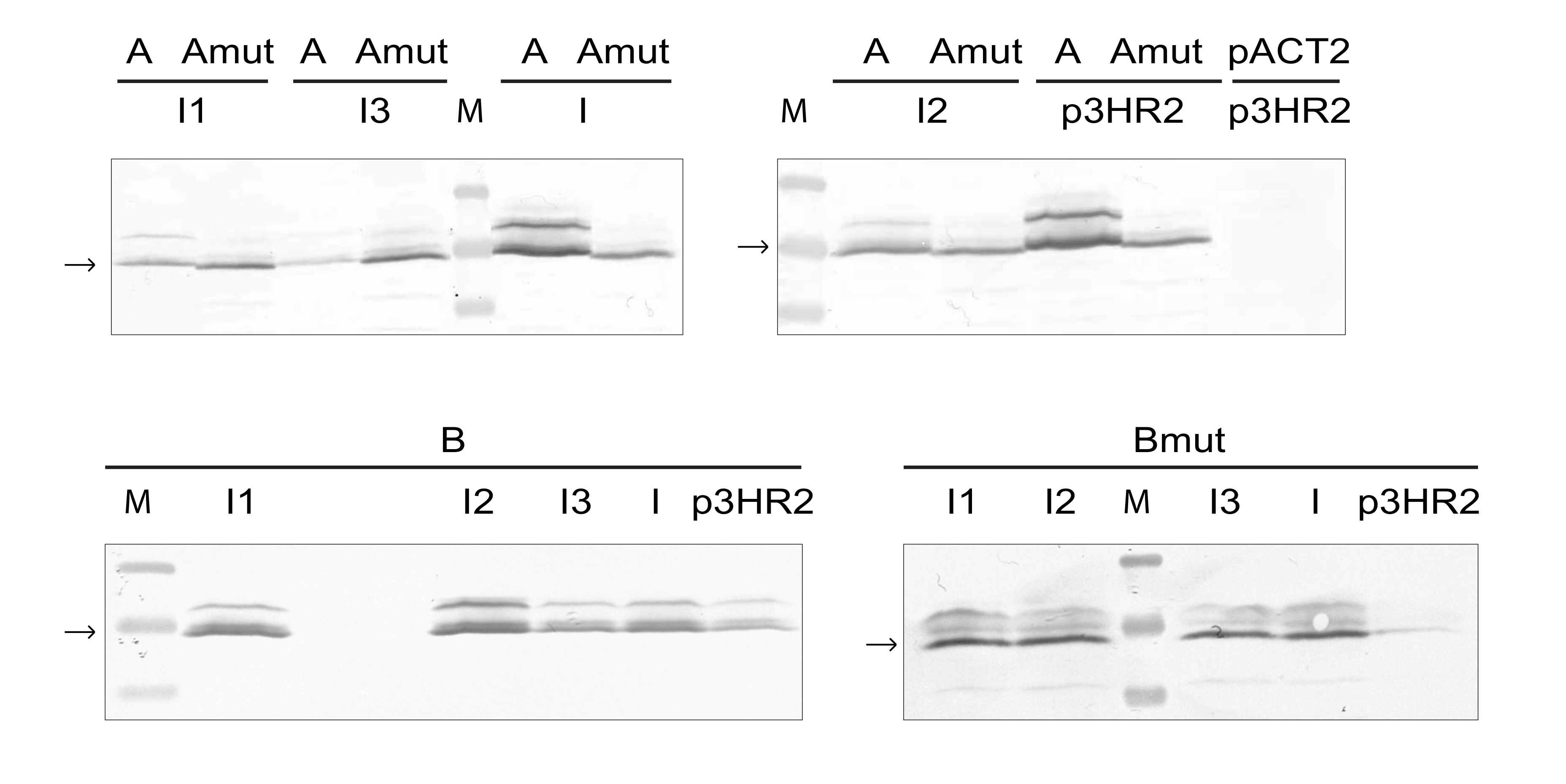

Supplement: S6 Fig — The production of Rpl22A, Rpl22B and their RNA binding mutants, fused with HA-Gal4 activation domain, was verified using Western blot analysis with anti-HA antibody. Hybrid proteins of 31 kDa (marked with arrow) were detected in extracts from all strains tested in three-hybrid system. M—protein size marker. (TIF) [file pone.0190685.s006.tif]
