# Supplementary material for: Introns provide a platform for intergenic regulatory feedback of RPL22 paralogs in yeast
Source: PLoS One. 2018 Jan 5;13(1):e0190685. doi: 10.1371/journal.pone.0190685 (PMC5755908; doi:10.1371/journal.pone.0190685)
Supplement: S6 Table — (PDF) [file pone.0190685.s012.pdf]

S6 Table. Numeric rendering of heatmaps in Fig. 3A.

Relative endogenous *RPL22A* mRNA abundance

Overexpression	Empty vector			*RPL22A*				*RPL22B*				*RPL22A* mutated				*RPL22B* mutated			
	Fold change	s.d.	Replicates	Fold change	s.d.	Replicates	*P* value	Fold change	s.d.	Replicates	*P* value	Fold change	s.d.	Replicates	*P* value	Fold change	s.d.	Replicates	*P* value
WT	1	0.22	5	0.46	0.05	3	3.06E-02	0.44	0.2351	3	4.86E-03	1.08	0.2	3	6.60E-01	1.4	0.05	3	7.40E-02
AΔ	-	-	-	-	-	-	-	-	-	-	-	-	-	-	-	-	-	-	-
BΔ	1.28	0.20	3	0.56	0.12	3	1.12E-01	0.65	0.16	3	1.19E-02	0.88	0.06	2	1.54E-01	1.33	0.27	3	8.78E-01
AΔi	1.38	0.09	3	1.07	0.05	2	5.72E-01	1.15	0.15	2	6.53E-01	1.03	-	1	-	1.75	0.11	3	5.72E-01
AΔiBΔi	1.34	0.29	3	1.02	-	1	-	0.97	-	1	-	-	-	-	-	-	-	-	-
BΔi	0.79	0.07	3	0.38	0.02	2	5.30E-02	0.45	0.04	2	1.51E-01	0.95	0.08	2	-	0.79	0.02	2	-

Relative endogenous *RPL22B* mRNA abundance

Overexpression	Empty vector			*RPL22A*				*RPL22B*				*RPL22A* mutated				*RPL22B* mutated			
	Fold change	s.d.	Replicates	Fold change	s.d.	Replicates	*P* value	Fold change	s.d.	Replicates	*P* value	Fold change	s.d.	Replicates	*P* value	Fold change	s.d.	Replicates	*P* value
WT	1	0.18	5	0.14	0.02	3	8.86E-05	0.14	0.03	3	5.22E-05	0.96	0.35	3	7.79E-01	1.33	0.35	3	4.06E-01
AΔ	14.41	0.31	3	1.10	0.52	3	5.04E-02	0.68	0.53	3	5.04E-02	11.78	3.2	3	2.35E-01	10.89	2.01	3	1.09E-01
BΔ	-	-	-	-	-	-	-	-	-	-	-	-	-	-	-	-	-	-	-
AΔi	0.09	0.01	3	0.07	0.03	2	1.00E+00	0.07	0.02	2	1.00E+00	0.11	-	1	-	0.09	0.01	3	1.00E+00
AΔiBΔi	5.10	0.32	3	5.37	-	1	-	3.98	-	1	-	-	-	-	-	-	-	-	-
BΔi	5.93	2.18	3	4.67	0.29	2	1.00E+00	4.49	0.56	2	1.00E+00	6.2	0.71	2	1.00E+00	6.11	0.37	2	1.00E+00

s.d. stands for standard deviation.

P values were produced by t-test with Holm correction for multiple testing, comparing dCt values of "Empty vector" samples to each of the overexpressing strains.
