# Supplementary material for: Introns provide a platform for intergenic regulatory feedback of RPL22 paralogs in yeast
Source: PLoS One. 2018 Jan 5;13(1):e0190685. doi: 10.1371/journal.pone.0190685 (PMC5755908; doi:10.1371/journal.pone.0190685)
Supplement: S7 Table — (PDF) [file pone.0190685.s013.pdf]

S7 Table. Numeric rendering of heatmaps in Fig. 4.

Relative *RPL22A* mRNA abundance

Overexpression	Empty vector			RPL22A				RPL22B			
	Fold change	s.d.	Replicates	Fold change	s.d.	Replicates	*P* value	Fold change	s.d.	Replicates	*P* value
AA-BB	1.00	-	-	0.42	0.05	3	4.69E-02	0.50	0.08	3	4.69E-02
BA-BB	0.98	0.09	6	0.07	0.01	6	3.29E-07	0.12	0.05	5	2.59E-06
AA-AB	0.92	0.33	6	0.49	0.10	6	9.99E-03	0.44	0.07	6	9.99E-03
BB-BB	-	-	-	-	-	-	-	-	-	-	-

Relative *RPL22B* mRNA abundance

Overexpression	Empty vector			RPL22A				RPL22B			
	Fold change	s.d.	Replicates	Fold change	s.d.	Replicates	*P* value	Fold change	s.d.	Replicates	*P* value
AA-BB	1.00	-	-	0.07	0.02	2	6.67E-02	0.08	0.00	2	1.84E-02
BA-BB	3.10	0.16	4	0.24	0.03	4	1.29E-05	0.34	0.06	3	1.28E-04
AA-AB	2.09	0.35	4	0.89	0.14	4	8.60E-03	1.25	0.37	4	2.36E-02
BB-BB: locus B	4.56	0.58	3	0.44	0.12	3	8.56E-04	0.57	0.25	3	4.38E-03
BB-BB: locus A	5.39	0.96	2	0.41	0.07	2	3.74E-02	0.44	0.02	2	3.74E-02

s.d. stands for standard deviation.

P values were produced by t-test with Holm correction for multiple testing, comparing dCt values of "Empty vector" samples to each of the overexpressing strains.
