# Supplementary material for: Introns provide a platform for intergenic regulatory feedback of RPL22 paralogs in yeast
Source: PLoS One. 2018 Jan 5;13(1):e0190685. doi: 10.1371/journal.pone.0190685 (PMC5755908; doi:10.1371/journal.pone.0190685)
Supplement: S1 File — (DOCX) [file pone.0190685.s016.docx]

**Supplementary methods**

**Verification of changes in mRNA expression in RPG intron deletion strains**

For the verification of intergenic regulation between *RPL2*, *RPL16*, *RPL17*, *RPL22*, *RPL37*, *RPS0* and *RPS18* paralogs, we prepared haploid strains with combinations of RPG alleles with/without intron from JPY156C3, JPY166B2, JPY166B7, JPY156B8, JPY156A9, JPY165I5 and JPY156A5 diploids (kindly provided by J. Parenteau), respectively, by random spore analysis. We confirmed strain identity by growth on selection media and by PCR genotyping. All other steps were performed as described in Methods using the same primers as in [3].

**Preparation of RNA degradation pathway/intron deletion double mutant strains and RNA degradation pathway mutants in combination with *prp45*(1-169)**

To prepare double mutant strains harboring combinations of *RPL22* intron deletions with concurrent deletion of genes from RNA degradation pathways, we crossed strains KAY61 and KAY67 with relevant strains from the yeast deletion collection [12]. After sporulation, we used random spore analysis to obtain the required double mutant strains, and we verified strain genotype by growth on selection media, ability to cross with BY4742 or BY4741, and by PCR with primers KA96 + KA97 for *RPL22A* and KA98 + KA99 for *RPL22B*.

Strains with deletion of an RNA degradation pathway component in combination with *prp45*(1-169) were prepared by the truncation of *PRP45* in strains bearing the desired deletion obtained from the yeast deletion collection [12]. The truncation was constructed by knocking in an integration cassette prepared by PCR using primers KM09 + KM10 and the pFA6a-3HA-NatMX6 plasmid as template [13]. Integration was confirmed by PCR with primers AV17 + AV18 and the expression of the truncated protein was verified by Western blotting using anti-HA antibody (HA.11; Covance).

**SDS-PAGE and Western blotting**

Cell pellets from 4.5 ml of mid-exponential cultures were resuspended in 100 μl of 1.85 M NaOH with 7% beta-mercaptoethanol, vortexed for 2 min and incubated on ice for 10 min. Then, proteins were precipitated by the addition of 100 μl of 50% TCA and incubated on ice for 5 min. Proteins were sedimented by centrifugation (13000 g, 10 min), neutralized with 500 μl unbuffered 1 M Tris, and dissolved in 100 μl of 2x Laemmli buffer. For analysis of 6xHIS-tagged Rpl22 proteins, 18 μl of protein samples were separated by Tris-tricine SDS-PAGE on a 10% gel. For analysis of the Gal4-HA-Rpl22 fusion, 15 μl of samples were separated on 12% Tris-glycine SDS-PAGE. Proteins were transferred to a nitrocellulose membrane using a BioRad wet Western blotting apparatus (2 hours at 115 V) and transfer buffer (25 mM Tris, 192 mM glycine and 20% methanol). Membranes were blocked with 5% blocking reagent (BioRad) in TBS with 0.05% Tween-20. To detect HIS and HA tags, anti-HIS (HIS.H8, Invitrogen) or anti-HA (HA.11, Covance) antibodies were diluted 1000 times in blocking solution and membranes were incubated for 1 hour at room temperature followed by overnight incubation at 8 °C. As a loading control, a section of each membrane was incubated with anti-PSTAIR antibody (P7962, Sigma) diluted 3000 times in blocking solution. Incubation with secondary Goat Anti-Mouse IgG conjugated with alkaline phosphatase (GAM-AP, BioRad) was performed at room temperature for 1 hour. Development was done using the AP Conjugate Substrate Kit (BioRad).

**Supplementary References**

1. Lorenz R, Bernhart SH, Höner zu Siederdissen C, Tafer H, Flamm C, Stadler PF, et al. ViennaRNA Package 2.0. Algorithms Mol Biol. 2011;6: 26. doi:10.1186/1748-7188-6-26

2. Gahura O, Abrhámová K, Skružný M, Valentová A, Munzarová V, Folk P, et al. Prp45 affects Prp22 partition in spliceosomal complexes and splicing efficiency of non-consensus substrates. J Cell Biochem. 2009;106: 139–151. doi:10.1002/jcb.21989

3. Parenteau J, Durand M, Morin G, Gagnon J, Lucier J-F, Wellinger RJ, et al. Introns within Ribosomal Protein Genes Regulate the Production and Function of Yeast Ribosomes. Cell. 2011;147: 320–331. doi:10.1016/j.cell.2011.08.044

4. Hook B. RNA-protein interactions in the yeast three-hybrid system: Affinity, sensitivity, and enhanced library screening. RNA. 2005;11: 227–233. doi:10.1261/rna.7202705

5. Heinisch JJ, Buchwald U, Gottschlich A, Heppeler N, Rodicio R. A tool kit for molecular genetics of Kluyveromyces lactis comprising a congenic strain series and a set of versatile vectors: Kluyveromyces lactis molecular tool kit. FEMS Yeast Res. 2010;10: 333–342. doi:10.1111/j.1567-1364.2009.00604.x

6. Převorovský M, Hálová M, Abrhámová K, Libus J, Folk P. Workflow for Genome-Wide Determination of Pre-mRNA Splicing Efficiency from Yeast RNA-seq Data. BioMed Res Int. 2016;2016: 1–9. doi:10.1155/2016/4783841

7. Siatecka M, Reyes JL, Konarska MM. Functional interactions of Prp8 with both splice sites at the spliceosomal catalytic center. Genes Dev. 1999;13: 1983–1993.

8. Melcher K. A Modular Set of Prokaryotic and Eukaryotic Expression Vectors. Anal Biochem. 2000;277: 109–120. doi:10.1006/abio.1999.4383

9. SenGupta DJ, Zhang B, Kraemer B, Pochart P, Fields S, Wickens M. A three-hybrid system to detect RNA-protein interactions in vivo. Proc Natl Acad Sci. 1996;93: 8496–8501.

10. Stumpf CR, Kimble J, Wickens M. A Caenorhabditis elegans PUF protein family with distinct RNA binding specificity. RNA. 2008;14: 1550–1557. doi:10.1261/rna.1095908

11. Gahura O, Hammann C, Valentová A, Půta F, Folk P. Secondary structure is required for 3’ splice site recognition in yeast. Nucleic Acids Res. 2011;39: 9759–9767. doi:10.1093/nar/gkr662

12. Winzeler EA, Shoemaker DD, Astromoff A, Liang H, Anderson K, Andre B, et al. Functional characterization of the S. cerevisiae genome by gene deletion and parallel analysis. Science. 1999;285: 901–906.

13. Van Driessche B, Tafforeau L, Hentges P, Carr AM, Vandenhaute J. Additional vectors for PCR-based gene tagging in Saccharomyces cerevisiae and Schizosaccharomyces pombe using nourseothricin resistance. Yeast. 2005;22: 1061–1068. doi:10.1002/yea.1293

14. Edgar RC. MUSCLE: multiple sequence alignment with high accuracy and high throughput. Nucleic Acids Res. 2004;32: 1792–1797. doi:10.1093/nar/gkh340

15. Sievers F, Wilm A, Dineen D, Gibson TJ, Karplus K, Li W, et al. Fast, scalable generation of high-quality protein multiple sequence alignments using Clustal Omega. Mol Syst Biol. 2014;7: 539–539. doi:10.1038/msb.2011.75
