# Supplementary material for: Introns provide a platform for intergenic regulatory feedback of RPL22 paralogs in yeast
Source: PLoS One. 2018 Jan 5;13(1):e0190685. doi: 10.1371/journal.pone.0190685 (PMC5755908; doi:10.1371/journal.pone.0190685)
Supplement: S2 File — (DOCX) [file pone.0190685.s017.docx]

Intron CLUSTAL multiple sequence alignment by MUSCLE (3.8)

K.l._RPL22 GTATGT----------------------ACAAGAAT---------GTTGTAAAATCGCTT

S.c._RPL22A GTATGATACTTT---------------AAAAGGAATCCACGCCCGATTACGAAGTC----

S.c._RPL22B GTACGTTAATTTTCGTTCCTATGTCACAATTAGAGAACATGAAAGAAAATAAAATC----

*** * * ** ** **

K.l._RPL22 GTTTTTAGAAGTGAATTGGTTACT-------ATTTGCTAGAATTATAACATCAGAGAATT

S.c._RPL22A --T-----ATGTTTTACTGTTATTGTAATG-ATTGATTGTACGCGAATAACAAGGAAGAT

S.c._RPL22B --TTTAAAAAGTTTGTCTATTCCTACAGTTCATTGAACAGTCCCGCTTTCACAGCGAACT

* * ** ** * *** ** * *

K.l._RPL22 TGAATATATTTCGTAAAGGATGAAGGACTGT---AAACCAACGACTTCCTCTGTATCGTC

S.c._RPL22A AGTAAAACCAAAAAAAAAAAAATAAAAATGTACGAAACGAAGAAGCGTCTATGTGT----

S.c._RPL22B AGTGGATTCCATCTCGAAGTTACA---------------------TTTTTATGTAT----

* * * * * *** *

K.l._RPL22 TCAAACAAGATT-TGGATGACAGAAGTTTCTAACAATGGAAAACTCAACTTGGTGGCTAT

S.c._RPL22A TTAGTTGCTTTTCTGTATTTTAAAAATT--CACTACTTGAACATTGAACTCTATATCGAC

S.c._RPL22B TCAGCTA---TTCTGTATTTTAAAAATT--CACCCCTTATAGCTTTAAC--AGAGGCAAT

* * ** ** ** * ** ** * * * * *** * *

K.l._RPL22 TCTGTTTTTTAATTATTCATCTTTAT---------TCAGCAATTG------ATGATTGTT

S.c._RPL22A TCCCTTCTTTTTATCCGTTTCTGAATGTTTTTTTGTTTGAGATTGTCATATGTGATTGTT

S.c._RPL22B GCGCTTTCTC---TAGCTATATGAA--------------------------GTGATTGTT

* ** * * * * * ********

K.l._RPL22 AATAGCAGTAGGCTGGTTTATTTTTTTTGTACAAGAGATTGTGTGGACTGCATACCTCAT

S.c._RPL22A AATAGCAGTAGGCAACTTTGTGGTTTCGGGATTGAA------------------------

S.c._RPL22B AATAGCAGTAGGCCAGACATTTTTTCCTCGTCCAAA------------------------

************* * ** *

K.l._RPL22 AGCAGCTTCAACAGATCAGTCGACCGATCTGTCTCTAGCTTCAAAGAGTGTATGCTATTC

S.c._RPL22A -----------------------------------------AGAAAATCGTGAAATATTC

S.c._RPL22B -----------------------------------------CGACAAGGATGAT---TTC

* * * ***

K.l._RPL22 AGTTCACATAGCTTGATTGACAAAGCAATGCCCTCCCTTGGACTTTGATTTACTAACGTT

S.c._RPL22A GTTTT--------------------------------------------TTACTAACATT

S.c._RPL22B ATCTC------------------------------------AGTTGGGATTACTAACATA

* ******** *

K.l._RPL22 AAAGCCATTCATCAAATTGAACGTTCGAATGTTCTTTTTGATTTGATCTCTATTCATACA

S.c._RPL22A ACTGTT----------CTGAAAATTTTTACTAATTTTCAATCTTTAATTTTTTTGATATA

S.c._RPL22B AAA-------------CTGTA------------------------------------ACA

* ** * * *

K.l._RPL22 G

S.c._RPL22A G

S.c._RPL22B G

*

Protein CLUSTAL O(1.2.4) multiple sequence alignment

S.c._Rpl22B MAPNTSRKQKVIKTLTVDVSSPTENGVFDPASYSKYLIDHIKVDGAVGNLGNAIEVTEDG

K.l._Rpl22 MAPNTARKQKITKTFTVDVSSPTENGVFDPASYAKYLIDHIKVEGHVGNLGQAITVEEDG

S.c._Rpl22A MAPNTSRKQKIAKTFTVDVSSPTENGVFDPASYAKYLIDHIKVEGAVGNLGNAVTVTEDG

*****:****: **:******************:*********:* *****:*: * ***

S.c._Rpl22B SIVTVVSSAKFSGKYLKYLTKKYLKKNQLRDWIRFVSIRQNQYKLVFYQVTPEDADEEED

K.l._Rpl22 SVVTIVSTTKFSGKYLKYLTKKYLKKNQLRDWIRFVSTKTNEYKLAFYQITPEDEEEEED

S.c._Rpl22A TVVTVVSTAKFSGKYLKYLTKKYLKKNQLRDWIRFVSTKTNEYRLAFYQVTPEEDEEEDE

::**:**::**************************** : *:*:*.***:***: :**::

S.c._Rpl22B DE

K.l._Rpl22 EE

S.c._Rpl22A E

:

**S2 File. Alignment of *RPL22* introns and proteins from *S. cerevisiae* and *K. lactis.***

*RPL22* intronic sequences were aligned using CLUSTAL multiple sequence alignment by MUSCLE (3.8) [14]. Identical nucleotides are marked by asterisks and the conserved regions are underlined. Protein sequence alignments were performed using Protein CLUSTAL O (1.2.4) multiple sequence alignment [15]. Identical amino-acids are marked by asterisks and conservative differences by colons and periods. Lysines that were changed to glutamates to prepare RNA binding defective mutants are shown in red.
